# How time horizons of autocrats impact health expenditure: a mixed methods research

**DOI:** 10.1186/s12889-020-08821-3

**Published:** 2020-05-11

**Authors:** Huang-Ting Yan, Yu-Chun Lin

**Affiliations:** 1 Department of Government, University of Essex, Colchester, UK; 2 Department of Chinese Medicine, China Medical University Hospital, No. 2, Yude Road, North District, Taichung City, Taiwan 40447

**Keywords:** Armed conflict, Dictator, Health expenditure, Synthetic control methods, Time horizons

## Abstract

**Background:**

A country’s spending on healthcare significantly improves its population health status. No comparative study has examined how the threat perceived by leaders influences health expenditure and cross-national analyses of authoritarian regimes. The objectives of this study are to examine how time horizons of autocrats influence health expenditure.

**Methods:**

We designed a mixed methods research approach. First, the study used panel data from 1995 to 2010 covering 95 countries (*n* = 1208) and applied fixed effects regression models. As a proxy for time horizons, the study generated the predicted survival time for each regime-year using parametric survival analysis and the predictors to model regime failure. Second, we chose Chad, Rwanda and Ivory Coast to apply synthetic control methods for comparative case studies. Armed conflict had significant effects on regime duration and was used for an intervention. We constructed a synthetic version of each country, combining counties that did not or did experience armed conflict to resemble the values of health expenditure predictors for the actual country prior to the intervention.

**Results:**

We found that an increase in the natural log form of survival time by 1 resulted in a 1.14 percentage point increase in health expenditure (% of GDP) (1.14, 95% CI = 0.60–1.69). Furthermore, we found that the difference in health expenditure between the actual Chad and its synthetic version starts to grow following the civil war in 2004 (in 2004, actual: 5.72%, synthetic: 5.91%; in 2005, actual: 3.91%, synthetic: 6.74%). Similarly, a large health expenditure gap between the actual Rwanda and its synthetic control resulted after the peace deal was signed in 2002 (in 2002, actual: 4.18%, synthetic: 4.77%; in 2003, actual: 6.34%, synthetic: 5.03%). In Ivory Coast, the two series diverge substantially during the civil war from 1999 to 2005 (in 1998, actual: 7.30%, synthetic: 7.11%; in 2002, actual: 4.47%, synthetic: 7.43%; in 2007, actual: 6.35%, synthetic: 6.50%).

**Conclusions:**

The findings suggest that health expenditure decreases as regime time horizons shrink, and reducing armed conflict is a way to promote regime stability.

## Background

A country’s expenditure on healthcare can significantly improve the health of its population [1, 2]. Recent research has examined the determinants of health expenditure from the perspective of political economy; discussing themes such as political regime types and transitions [3, 4], post-socialist restructuring of health funding and management patterns [5, 6], the ideology of governing parties and political competition [7, 8], fiscal decentralisation, [9, 10], and the representation of women in politics [11, 12]. However, no comparative study has examined how the threat perceived by leaders influences health expenditure and cross-national analyses of authoritarian regimes.

Recent literature argues that rulers in a multi-party system transfer spending from health to deal with the opposition because multiparty elections give regime outsiders an opportunity to challenge authoritarian incumbents and, thus, are dangerous to the regime [4]. Multiparty election, however, is not a single source of authoritarian instability. Literature also highlights the effects of regime types, economic performance and social diversity on a regime’s stability [13–15]. Thus, we must develop a more holistic approach in combining political, economic, and social factors for regime failure that can better reflect the level of perceived threat and, subsequently, the time horizon of autocrats.

A dictatorship occurs when the chief executive is chosen in a regularised selection process within the political elite and, once in office, the autocrat exercises power with few institutional constraints. Time horizons of autocrats refer to how long rulers expect to remain in power. A ruler’s expectation of the length of this duration is strongly associated with levels of health expenditure. At least four causal mechanisms might explain this possible link.

The first mechanism, derived from research on the corruption of dictatorships [16, 17], posits that authoritarian regimes of shorter duration are generally more corrupt because regime instability causes autocrats to develop a short time horizon that increases their willingness to expropriate any capital asset for personal wealth. Thus, a decline in national income reduces their tax collection in the long run, followed by decreased government spending. There is also evidence that corruption reduces spending on health [18, 19].

An analysis of how dictators deal with an imminent threat points to the second causal mechanism. The risk of a coup causes leaders to increase spending on militarised interstate disputes [20] and support for pro-government mobilisation [21]. Opposition protests facilitate the incumbent’s use of repression against radical claims [22], with the government reallocating budget away from social programmes to fund a coercive response. Recent research has indicated the incumbent’s response to anti-government violence through budgetary shifts from health to defence for stopping an emerging threat [23]. Further, dictators holding multiparty elections tend to transfer government spending on healthcare to reduce opposition threats [4].

A third explanation can be derived from studies of state strength, which suggest that state power promotes authoritarian durability to actively administer their subjects and territory [24], implying that the shorter the rulers expect to remain in power, the lower the possibility that the state has sufficient institutional capacity to levy taxes and provide public goods. Scholars found that a high political instability in autocracies can be detrimental to the population health status [25], which may be related to health expenditure levels. First, armed conflict or violence can disrupt the Leviathan in providing healthcare. Second, foreign investment significantly alters the expenditure composition in favour of social spending [26] and tends to decline in an uncertain political environment. Third, political instability increases the government’s incentive to seek foreign aid, which is fungible with domestically-financed government spending on health, thus decreasing government health expenditure [27].

Finally, short-lived regimes decrease the dictator’s incentive to maintain social stability (e.g. creating a strong social safety net), which may increase the risk that citizens will experience social upheaval, employment instability, and other hardships. It is likely that people will then sense a bleak prospect under which they value current benefit much more than that in the future, resulting in people reducing investment in health insurance and expenses for important medical services in exchange for other expenditures. One study found that housing instability and food insecurity are associated with poor health care access [28], while another suggests that families under income instability potentially skip or delay health care [29].

The aim of this study is to examine the determinants of health expenditure in dictatorships. Based on previous studies, we hypothesised that the time horizons of autocrats exert a positive effect on a country’s health expenditure. Using a mixed research combing panel data analysis with synthetic control methods for comparative case studies, we found supporting evidence in the case of the ruler’s time horizons for these arguments. These findings have significant policy implications that health expenditure decreases as regime time horizons shrink, and reducing armed conflict is one of the ways to promote regime stability.

## Methods

The unit of analysis in this study was ‘a dictatorship’, using the Polity IV Project as the main source of measures. The Polity IV data series scales regimes from − 10 to + 10, and we identified dictatorships as regimes with scores lower than 6 (< 6), screening all countries between 1995 and 2010.

Health expenditure was defined as the total spend in the country on health as a proportion of Gross Domestic Product (GDP), using data from the World Bank. This research uses the predicted regime duration as a proxy for autocratic time horizons, instead of using regime interruptions by coups in the previous year, the magnitudes of armed conflicts, years in office for the chief executive, and the actual regime duration as proxy variables. We generated the predicted regime duration for each regime-year using the following predictors to model survival time: GDP per capita, ethnic and religious fractionations, the magnitudes of armed conflict, and a list of regime-type variables. We employed the parametric survival model in which survival time is assumed to follow an exponential distribution. Control variables that influence health expenditure were added, including prevalence of HIV [30] and multiparty elections [4]. A summary of the variables, operationalisation of indicators, and data sources is shown in Supplementary Table 1.

To investigate if the predicted regime duration corresponded to the real-world dynamics of regime change, the study provided some examples accounting for variation across and within regimes. Further, we tested the reliability of the proxy for autocratic time horizon by examining its correlation with other measures of the same underlying concept, such as regime interruptions by coups in the previous year, the magnitudes of armed conflicts, or the actual regime duration. Existing research posits that rulers who expect to remain in power for longer are less corrupt [16, 17], because an insecure autocrat with a higher likelihood of regime failure and shorter time horizon has an incentive to accumulate personal wealth, while autocrats with stable regimes have longer time horizons that allow them to promote good governance. We examined the validity of the predicted regime duration by exploring its effects in relation to the level of corruption in authoritarian polities (see Supplementary Data Analysis).

We used panel data from 1995 to 2010, covering 95 countries with a sample of 1208 pooled time series and cross-sectional observations. Fixed effects regression models were used, based on the results of the Hausman test. Further, we lagged the independent variable by one period to allow some time for its effect on health expenditure to materialise. For robustness tests, this study changed the threshold of the determinant factor, identifying regimes with Polity IV scores lower than 1 (< 1) as dictatorships. Thus, it excluded regimes with scores ranging from 5 to 1 because scholars do not agree that they are dictatorships. We also used the conventional method to operationalise the ruler’s expectation of remaining in power, (i.e. the actual regime duration), because autocrats often learn how to use a variety of ways to contain threats to ensure their survival, especially if they have stayed in power for some time. Finally, we generated the predicted probability of regime failures as a proxy for autocratic time horizons using the same predictors (e.g. GDP per capita) but through logistic regression with a cubic polynomial of time to control for time dependence.

This study also applies synthetic control methods for causal inference in comparative case studies. Compared to regression-based comparative case studies that rely on extrapolation to construct a comparison unit, the synthetic control method avoids extrapolation biases constructing a weighted average of unit [31]. In contrast to regression-based studies having a large enough sample for treatment and control groups, the synthetic control method can examine causal mechanisms based on a single treated unit and this is used in this study, given the small sample of the data.

Based on the results of the parametric survival model, some types of dictatorships, GDP per capita, religious fractionation, and armed conflict had significant effects on regime duration (see Supplementary Table 2). It is difficult to determine the threshold for the occurrence of an event of a continuous variable, such as economic development. In contrast, armed conflict could be used for constructing a specific event or intervention where 1 = major episodes of international, civil, and ethnic warfare involving the state, and 0 = no episodes. We could thus compare the outcomes between units representing the case of interest, defined by the occurrence of armed conflict, causing the dictator to sense a threat to the current regime and subsequently decrease the health expenditure, and otherwise similar but unaffected units.

We chose three cases of armed conflict: Chad experienced a civil war after 2004 that involved different forces, the Chadian and Sudanese governments, and the rebels in both countries; Rwanda, where a peace deal was signed with the Democratic Republic of the Congo in 2002 to end its involvement in the neighbouring country’s civil war from 1996; and Ivory Coast, where the violence since 2000 between Muslims and Christians escalated into a civil war in 2002 that ended in 2005 with the Pretoria Agreement. Even though there are relatively more comparative studies applying synthetic control methods, such countries that were exposed to an event only for 1 year (e.g. the Albanian civil unrest of 1997) or intermittently between 1996 and 2010 (e.g. a failed coup attempt, rebellion and violence in the Central African Republic between 2001 and 2003, and Bush War from 2005), do not help in estimating causal inference because authoritarian leaders may be able to avoid making timely adjustments in health spending if left with short periods of regime. In contrast, our three cases, each having at least 5 years of armed conflict and beginning in a different year during 1996–2010, have the advantage of having included robustness tests for time.

To find comparison units, we selected countries both with and without armed conflict between 1996 and 2010, from among those that can minimise the mean square error of the synthetic control estimator, based on their weighted average [31]. Supplementary Table 5 shows the weight of each country in the synthetic control. Supplementary Table 4 compares the characteristics of the three countries of interest, prior to the intervention to those of the individual synthetic version, suggesting that the synthetic counterpart is very similar to the actual one in terms of GDP per capita, multiparty elections, and one-year lag of health expenditure, providing a better counterfactual of interest.

We run a robustness check to test the sensitivity of our results to more synthetic control estimators or changes in the country weights. First, the study used a wide set of predictors: per capita GDP, one-year lag of health expenditure, multiparty elections, prevalence of HIV, constitutional system and dominant party regimes. Second, the study constructed a synthetic country excluding in each iteration one of the countries that received a positive weight in the original model (see Supplementary Tables 4 and 5). We further examined if the results are indeed indicative of the negative effects of armed conflict and not driven by unobservable factors, reassigning the treatment of interest in the data to 1 year before the actual event. We also reassigned the treatment in the data to a comparison unit. If the theoretical expectations were true, the ratio of the post-intervention to pre-intervention health expenditure gap between the case of interest and its synthetic counterpart would be greater than 1, indicating that the intervention had a large effect. In contrast, the corresponding ratio for the gap between the comparison unit and its synthetic control was relatively small. However, a large post-intervention gap does not indicate a large intervention effect unless health expenditure changed in the predicted direction. Thus, we examined if the estimated effect for the case was unusually large relative to the distribution of placebo effects and was in the predicted direction.

## Results

**Fig. 1:**
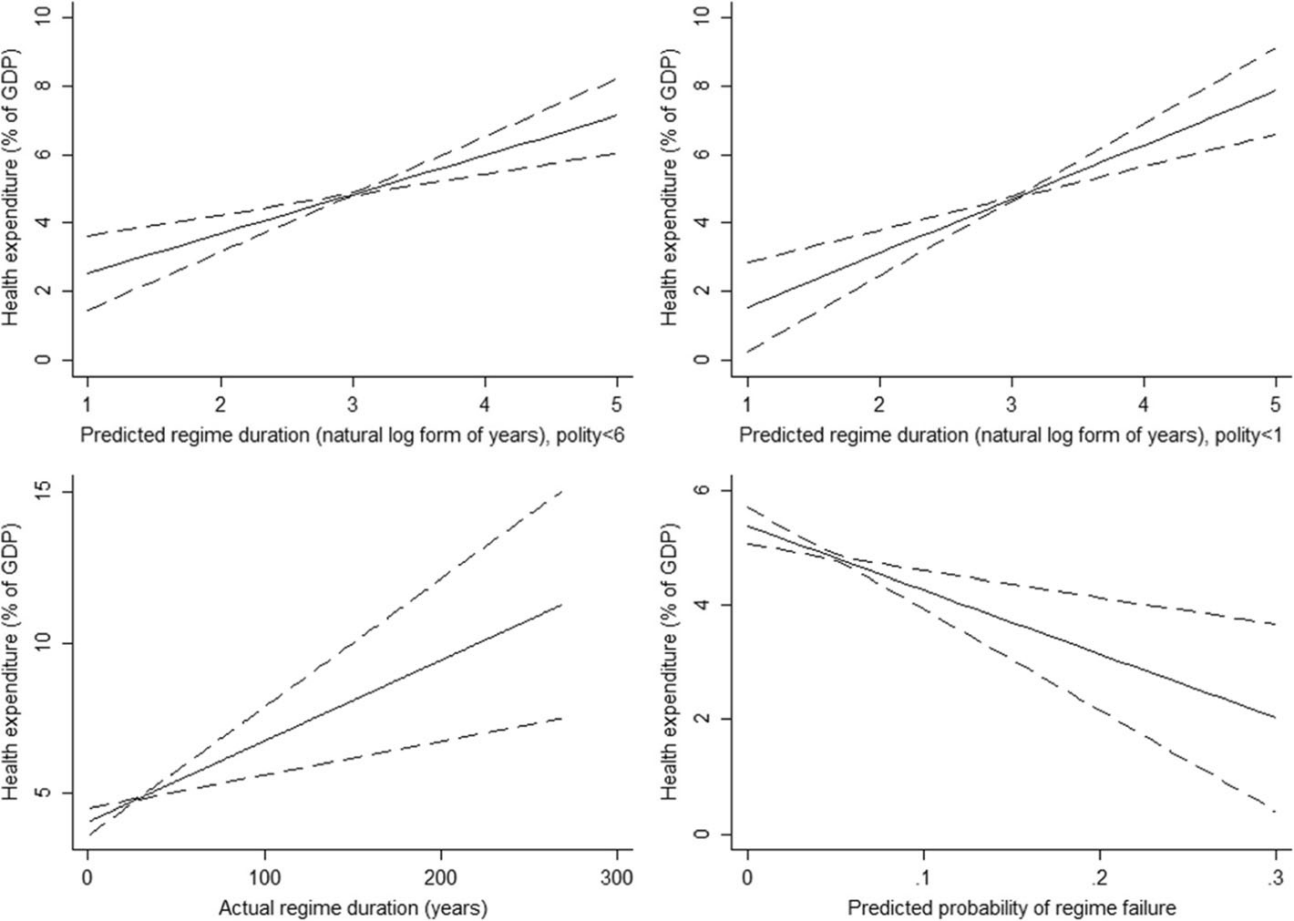
Authoritarian Time Horizon and Health Expenditure, 1995–2010. Note: regression coefficient: upper-left panel: 1.143***, 95% CI = [0.600, 1.686]; upper-right panel: 1.576***, 95% CI = [0.932, 2.219]; lower-left panel: 0.027***, 95% CI = [0.011, 0.043]; lower-right panel: − 11.206***, 95% CI = [− 17.744, − 4.668], * *p* < 0.10, ** *p* < 0.05, and *** *p* < 0.01. All results were based on panel data analysis with country and time fixed effects. Results illustrated in upper-left, upper-right, lower-left and lower-right panel correspond to model 1, 2, 3 and 4 of Supplementary Table 3. Source: the author

Figure 1 presents a positive linear relationship between the time horizons of the ruler and the level of health expenditure. For example, an increase in the natural log form of survival time by 1 resulted in a 1.14 percentage point increase in health expenditure (%) (1.14, 95% CI = 0.60–1.69). Our theoretical expectations were confirmed when this study changed the threshold for the determinant of dictatorships (1.58, 95% CI = 0.93–2.22), used the actual regime duration as a proxy for time horizons (0.03, 95% CI = 0.01–0.04), or generated the predicted probability of regime failures (0–1) using logistic regression with a cubic polynomial of time (− 11.21, 95% CI = − 17.74–-4.67) (for details see Supplementary Table 3).

**Fig. 2:**
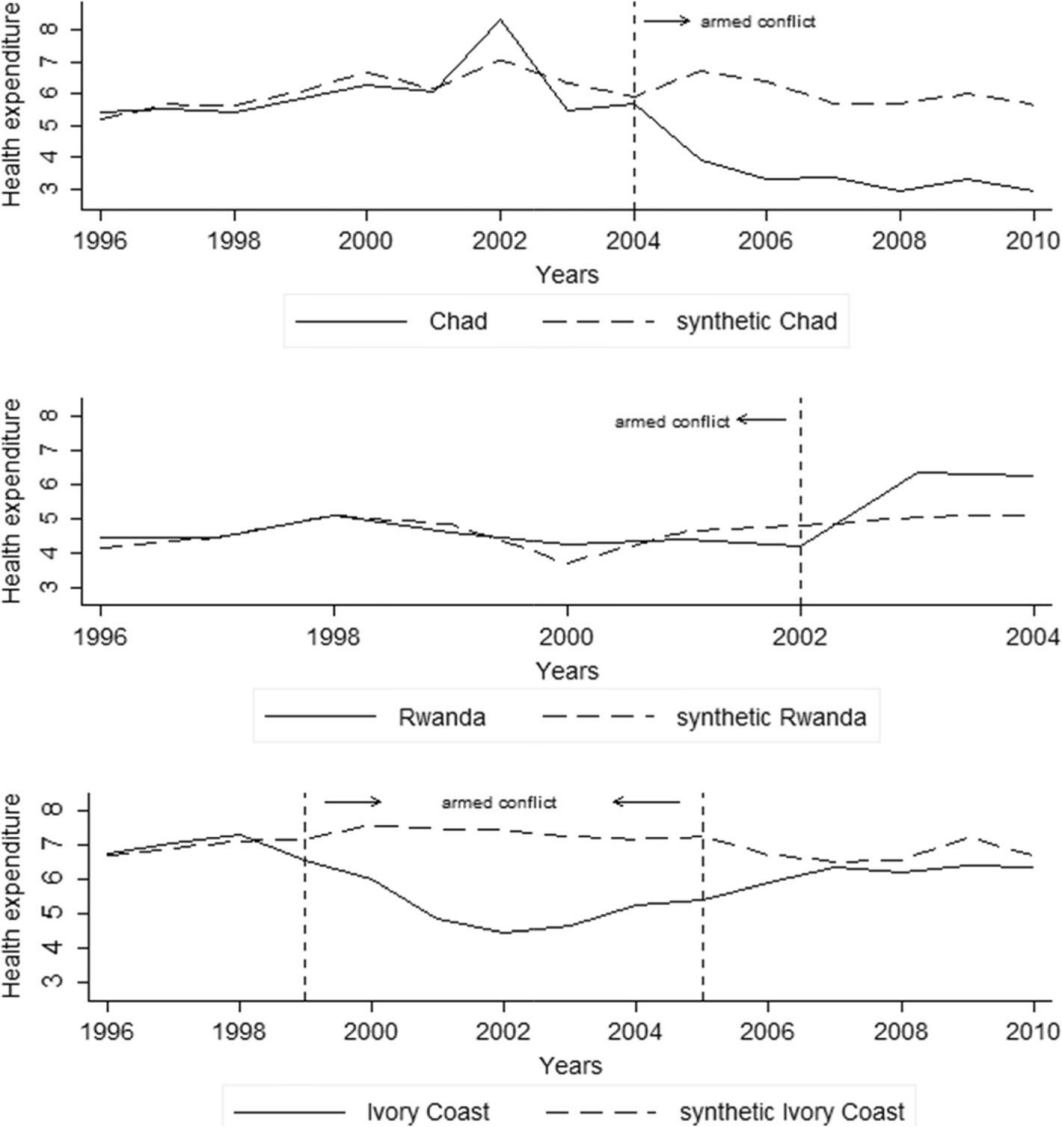
Trends in Health Expenditure: Actual country vs. Synthetic country. Note: health expenditure from 1996 to 2010 (upper panel): actual Chad (5.410, 5.557, 5.412, 5.866, 6.277, 6.086, 8.332, 5.491, 5.720, 3.910, 3.322, 3.346, 2.916, 3.307, 2.949) and synthetic Chad (5.215, 5.684, 5.620, 6.078, 6.696, 6.153, 7.075, 6.361, 5.917, 6.735, 6.408, 5.717, 5.686, 6.000, 5.657); health expenditure from 1996 to 2004 (middle panel): actual Rwanda (4.435, 4.417, 5.078, 4.573, 4.221, 4.380, 4.176, 6.342, 6.245) and synthetic Rwanda (4.145, 4.437, 5.079, 4.804, 3.689, 4.601, 4.774, 5.028, 5.107); health expenditure from 1996 to 2010 (lower panel): actual Ivory Coast (6.741, 7.066, 7.298, 6.522, 6.001, 4.846, 4.467, 4.652, 5.237, 5.390, 5.871, 6.346, 6.206, 6.414, 6.324) and synthetic Ivory Coast (6.687, 6.897, 7.109, 7.166, 7.579, 7.469, 7.431, 7.245, 7.159, 7.247, 6.715, 6.503, 6.557, 7.217, 6.684). Source: the author

Figure 2 demonstrates that the ‘synthetic’ Chad almost replicated health expenditure for the ‘actual’ Chad during the pre-treatment period from 1996 to 2004. The difference in health expenditure between the two series started to grow when a civil war broke out after 2004 in the actual Chad, while the synthetic Chad had a higher level of health expenditure (in 2004, actual: 5.72%, synthetic: 5.91%; in 2005, actual: 3.91%, synthetic: 6.74%). Similarly, a synthetic control that experienced the armed conflict closely approximated the health expenditure for Rwanda during the pre-treatment period from 1996 to 2002. The two series showed a large gap after the peace deal was signed in 2002 in the actual Rwanda, exhibiting a higher level of health spending (in 2002, actual: 4.18%, synthetic: 4.77%; in 2003, actual: 6.34%, synthetic: 5.03%). Finally, Ivory Coast first declined and later rose in terms of health expenditure, compared to its synthetic counterpart, which remained relatively constant in the outcome of interest. In other words, a synthetic control that did not confront armed conflict closely fit the economic characteristics of Ivory Coast before 1999 and after 2005. From 1999 to 2005, however, the two lines diverge substantially when the violence escalated into a civil war in the actual Ivory Coast during which the synthetic Ivory Coast had a higher level of health expenditure (in 1998, actual: 7.30%, synthetic: 7.11%; in 2002, actual: 4.47%, synthetic: 7.43%; in 2007, actual: 6.35%, synthetic: 6.50%). These findings confirm a pronounced adverse effect of armed conflict on health expenditure.

Our results were robust to a series of tests. Taking the case of Chad, the inclusion of more predictors (see Supplementary Figure 1 and Supplementary Tables 6 and 7) or changes in the country weights (see Supplementary Figure 2 and Supplementary Tables 8 and 9) did not change our results substantively. Furthermore, when a civil war was reassigned to a year earlier than the actual event, the health expenditure trajectories of Chad and its synthetic version did not diverge considerably in 2004 but did so in 2005 (in 2004, actual: 5.72%, synthetic: 6.01%; in 2005, actual: 3.91%, synthetic: 6.80%), marking out the actual event in 2004 as the determinant of the country’s health expenditure (see Supplementary Figure 3 and Supplementary Tables 10 and 11). We found that by reassigning the treatment to the comparison units in 2004 and obtaining their synthetic control estimates, the ratio of post-intervention to pre-intervention gap in health expenditure between other comparison units and their synthetic counterparts is small, except for in Djibouti, Gambia, and Swaziland (see Supplementary Figure 4). However, compared to Chad, the three units with a higher ratio displayed a different pattern of health expenditure gap after 2004: a negative gap for Chad (in 2005, − 2.83%) and positive gap for others (in 2005, Gambia: 0.05%; Djibouti: 1.36%; Swaziland: 2.05%) (see Supplementary Figure 5). This suggests that armed conflict exerts a negative effect on a country’s health spending.

## Discussion

Health expenditure included public and private spending on health, and time horizons of autocrats may be relevant to one of these components only. Based on the proposed mechanisms, the predicted regime duration should have a positive effect on health expenditure, either in public or private sources, as shown in Supplementary Figure 6. Existing research argues that economic crises could have serious consequences on public [32] and private health care expenditure [33], with evidence that civil wars have a significant impact on a country’s growth in GDP per capita [34], which may affect autocratic survival. This analysis controlled for the effect using the magnitudes of armed conflict as a predictor of expected regime duration. GDP per capita growth, however, may exert an independent effect on health spending. Supplementary Table 12 confirms our theoretical expectations including the additional control.

We also found a positive linear relationship between the autocrats’ expectations of regime duration and the level of health expenditure, with a positive effect on health spending probably only in stable authoritarianism. This is because leaders in unstable regimes face higher risks of irregular uprisings, and the risk of a coup causes leaders to increase government spending on militarised interstate disputes [20] and support pro-government mobilisation [21], thus creating a minimum level of health expenditure. Given a limited period of data for countries such as Iraq, Pakistan, and Thailand, this study studied those countries where authoritarianism survived the period between 1995 and 2010. Supplementary Table 13 confirms our theoretical expectations.

Existing research posits that the income elasticity of health expenditure varies with income level [35, 36], indicating that our results may differ according to a country’s income. Among low- and middle-income countries, BRICS and Next-11 nations have exhibited the strongest growth in total health spending [37, 38], while private health expenditure growth was recorded in Commonwealth of Independent States and Central Asian Republics Information Network nations [39]. A better approach could have been to divide 95 countries into four income groups, but fewer cases of upper-middle and high-income autocratic countries produced large parameter estimates and standard errors. The study, therefore, excluded countries based on their income level. Supplementary Figure 7 shows that the effect was more prevalent for low-income groups, implying that poverty-related diseases, such as malaria and tuberculosis, in poorer countries leads increased health spending on a large scale, once regime consolidation occurs. In Supplementary Figures 8 and 9, we reported additional robustness tests.

There is also a problem of reverse causality: health expenditure can reduce the opposition’s willingness to pursue revolutionary change. To eliminate endogeneity, the study developed an instrumental variable (IV); which is the percentage of other global democracies. A good IV should have a theoretical interpretation that it is expected to influence the endogenous variable but is unrelated to the outcome. This is because more democracies would increase opposition strength, leading to the autocrats’ co-optation efforts and institutional concessions and thus giving them a stake in their political survival [40]. However, it is theoretically implausible that regime dynamics of foreign countries relate to domestic health spending. Supplementary Figure 10 shows that the results from IV are similar to the Ordinary Least Squares (OLS) estimation, with estimated coefficients much larger than in OLS.

We also found that some comparison units (e.g. Gambia) did not closely fit the health expenditure pattern of their synthetic control estimates after the intervention of interest, suggesting alternative causal explanations in addition to our hypotheses. However, it is not explained by the prevalence of HIV [30] or political economy perspectives that an increase in women elected to politics is associated with increased government expenditures toward health [11], given that these countries have lower HIV incidence and female parliamentary representation. It is thus necessary to explore alternative causal mechanisms.

Finally, the findings show that multiparty elections and the prevalence of HIV had no significant impact on health expenditure. A possible explanation is that, in addition to prompting dictators to transfer government spending on healthcare to manage the opposition, elections enlarge the social basis in regimes where leaders previously rely on support from specific groups, thus encouraging more spending on healthcare [4]. Thus, multiparty elections are a double-edged sword. Low-to- middle-income countries bear the overwhelming burden of HIV in terms of the numbers of their citizens living with HIV, thus creating higher budgetary allocation of health budget on this. The potential to generate additional resources to fight HIV, however, is concentrated in some countries, with substantial variation characterising spending on HIV in high-prevalence countries [30].

The result covers several central themes in health economics and comparative politics. First, it is universally accepted that if a country goes through instability, the level of health expenditure tends to decrease. Existing literature, however, mainly examines whether political, economic, or social risks explain health spending [4, 23, 27]. This paper adds to existing scholarship, combining potential risk factors for regime failure that can reflect perceived threat (i.e. the time horizons of autocrats). Second, while scholars of political economy emphasise the importance of institutional characteristics in explaining government expenditure on health [3–12], there is lack of comparative work on how the autocrat’s time horizon influences health expenditure. Third, this research extends the unit of analysis to non-democratic regimes, beyond the scope of existing work that has been limited to OECD countries [7, 9, 10] or specific regions [5, 6, 8].

This study offers measurement of regime time horizons and a mixed research, combining panel data analysis with synthetic control methods for comparative case studies. This measure has several advantages over the current proxy for time horizons, as it allows for a combination of risk factors for regime collapse, per-year observations and different time horizons at the same regime age. Additionally, the use of synthetic control methods help in quantitative inference in small-sample studies [31]. The research design in this study addresses the limitations from most qualitative and quantitative approaches, thus improving validity.

A limitation of this study, however, is that countries were observed over too short a period to lead to causal inference, which is because of the limited database of the World Bank. The proxy for time horizons, the predicted regime duration, is influenced by the inclusion of different predictors to model regime failure, leading to another limitation. We found that the failure or survival rate of a regime was explained by nearly all predictors of our model (see Supplementary Table 2), thus avoiding the inclusion of irrelevant variables. We also examined the validity of a measure of time horizons, subjecting it to convergent validation, comparing with real-world cases, and exploring its performance in relation to existing theories proposing that corruption rises as regime horizons shrink. Finally, a model generating the predicted regime duration may be modified to exclude some observations. This is because dictators can evaluate how long they stay in power based on preceding observations and update their belief according to yearly observations. Thus, we risk using unobservable information if an empirical model of regime survival includes all observations, and future research could test the theoretical expectations as the model changes.

A key policy implication is that the efforts to drive the government and households to increase health expenditure could start with an increase in the regime’s time horizon. Further, reducing armed conflict is one of the ways to lower the uncertainty about the dictator’s term in office and, in turn, increase a country’s spending on health.

## Conclusions

The findings suggest that authoritarian regimes whose leaders face longer time horizons are associated with relatively high levels of health expenditure. Armed conflict may reduce regime duration, thus exerting a negative effect on the level of health expenditure.

## Supplementary information

**Additional file 1.** Supplementary Data Analysis.

**Additional file 2. Supplementary Table 1.** The Summary of Variables, Operationalisation of Indicators, Data Sources and Descriptive Statistics. ** Supplementary Table 2.** Authoritarian Regime Duration and Failure, 1945-2010. **Supplementary Table 3.** Authoritarian Time Horizon and Health Expenditure, 1995-2010. **Supplementary Table 4.** Health Expenditure Predictor Means. **Supplementary Table 5.** Country Weights in the Synthetic Country. **Supplementary Table 6.** Health Expenditure Predictor Means. **Supplementary Table 7.** Country Weights in the Synthetic Country. **Supplementary Table 8.** Health Expenditure Predictor Means. **Supplementary Table 9.** Country Weights in the Synthetic Country. **Supplementary Table 10.** Health Expenditure and Predictor Means. **Supplementary Table 11.** Country Weights in the Synthetic Country. **Supplementary Table 12.** Authoritarian Time Horizon and Health Expenditure, 1995-2010. **Supplementary Table 13.** Authoritarian Time Horizon and Health Expenditure, 1995-2010 (stable authoritarianism).

**Additional file 3. Supplementary Figure 1. ** Trends in Health Expenditure: More Predictors. **Supplementary Figure 2.** Trends in Health Expenditure: The Leave-one-out Estimates. **Supplementary Figure 3.** Placebo Armed Conflict-Trends in Health Expenditure: Actual Country vs. Synthetic Country. **Supplementary Figure 4. **Ratio of Pre-treatment RMSPE to Post-treatment RMSPE: Chad and Control Countries. **Supplementary Figure 5.** Health Expenditure Gap: Actual Country versus Synthetic Country. **Supplementary Figure 6.** Authoritarian Time Horizon and Health Expenditure, 1995-2010. **Supplementary Figure 7.** Authoritarian Time Horizon and Health Expenditure, 1995-2010. **Supplementary Figure 8.** Authoritarian Time Horizon and Health Expenditure, 1995-2010. **Supplementary Figure 9.** Authoritarian Time Horizon and Health Expenditure, 1995-2010. **Supplementary Figure 10.** IV Analysis: The Coefficient Plot of Predicted Regime Duration, 1995-2010.

## Data Availability

The datasets used and/or analysed during the current study are available from the corresponding author on reasonable request.

## Abbreviations

BRICS: Brazil, Russia, India, China and South Africa
CI: Confidence interval
GDP: Gross domestic product
HIV: Human immunodeficiency virus
IV: Instrumental variable
OECD: Organisation for Economic Co-operation and Development
OLS: Ordinary least squares
