# Supplementary material for: How time horizons of autocrats impact health expenditure: a mixed methods research
Source: BMC Public Health. 2020 May 11;20:649. doi: 10.1186/s12889-020-08821-3 (PMC7216651; doi:10.1186/s12889-020-08821-3)
Supplement: Supplementary file 1 — Additional file 1. Supplementary Data Analysis. [file 12889_2020_8821_MOESM1_ESM.docx]

**Supplementary Data Analysis**

This research presents evidence on the reliability of the proxy for autocratic time horizon. First, the study investigated if the predicted regime duration corresponded to the real-world dynamics of regime change. Figure 1 presents the predicted regime duration and probabilities of regime failure for selected countries, which shows temporal variation, and the cross-sectional difference between 1945 and 2010. The predicted failure probabilities for Singapore and Botswana were low, and as the real-world politics show, both dominant party regimes never fail. Regime failure rates were lower in Romania until 1989 when violent civil unrest substantially increased the risk of Communist regime collapse. Iran exhibited similar pattern to Romania, with a substantial increase in the predicted failure probabilities in 1979, reflecting a revolution that would result in the overthrow of the last monarch of Iran, Pahlavi. After that, a new Islamic regime led by Khomeini came to power. The other four countries suffered higher failure probabilities. DR Congo displayed a more volatile pattern: the regime remained stable after President Mobutu liquidated his political opponents in 1965 until the country faced economic deterioration and social unrest in 1990, which signalled the probability of regime failure. Subsequently, in 1997, the data show a large spike, which reflected that rebel forces led by Kabila expelled Mobutu from the country. By contrast, the five regimes in Pakistan, either personalist or military rule, faced relatively high failure rates and were toppled quickly.


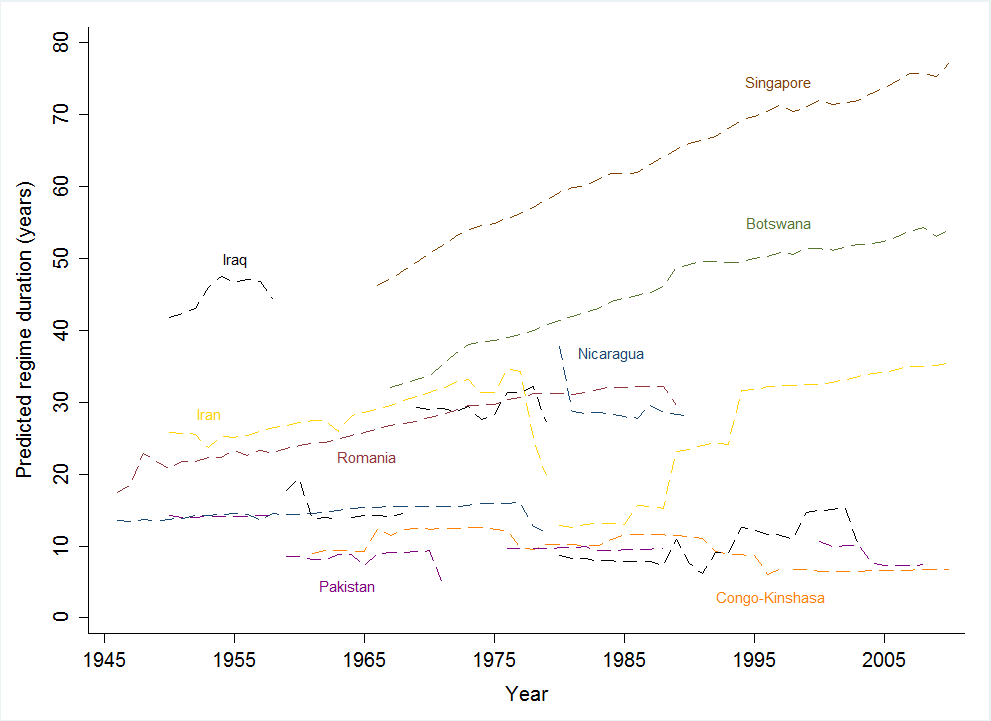


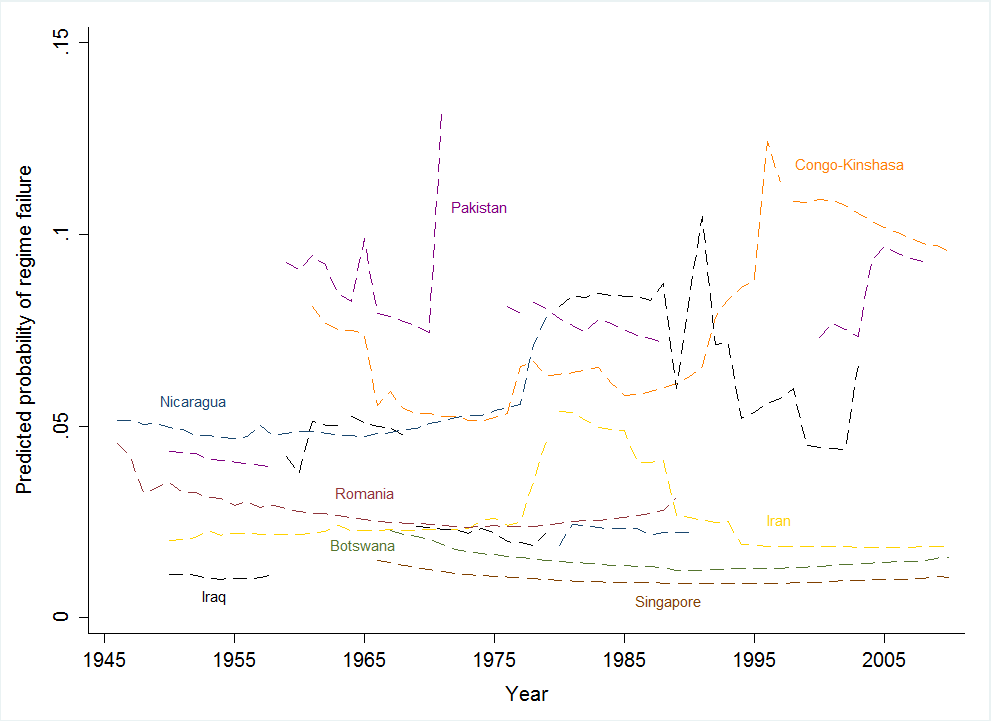


**Figures 1.** Authoritarian Time Horizons, 1945–2010

*Note*: depicts the predicted regime duration (upper panel) and the predicted probability of regime failure (lower panel) for selected countries, across time. The number of dictatorships varies across countries, for example, 5 in Iraq, 2 in Nicaragua and 1 in Botswana. *Source*: the author.

Second, we tested the reliability of the proxy for autocratic time horizon by examining its correlation with other measures of the same underlying concept, that was, regime interruptions by coups in the previous year [1], the magnitudes of armed conflicts [2], or the actual regime duration [3]. Table 1 found that the predicted regime duration (or probability of regime failure, see Wright’s seminal work [4]**)** was correlated with other measures of autocratic time horizons: regime interruptions by coups in the previous year (ρ = -.30, p < .001**)**, the magnitudes of armed conflicts (ρ = -.28, p < .001**)**, and the actual regime duration (ρ = .37, p < .001**)**.

Finally, we examined the validity of the predicted regime duration by exploring its effects in relation to the level of corruption in authoritarian polities. Our measure of the dependent variable was based on the Transparency International’s Corruption Perception Index (CPI), an indicator widely used in the cross-national literature [5]. The measure defines corruption as the abuse of public power for private gain, and provides the perceived level of public-sector corruption in 180 countries on a scale from 10 (least corrupt) to 0 (most corrupt). We selected 2008 because data for the year covered the most authoritarian regimes and included few missing values for other important variables. A list of control variables based on the comparative literature on corruption, including institutional factors [6], histories of British rule [7] and women’s representation in the parliament [8], was incorporated into our analyses. We expect corruption to be more frequent as the time horizon of the dictator shortens; a cross-sectional analysis was used to test the association.

**Table 1.** The Correlation Matrix of Measures of Autocratic Time Horizon

|  | Predicted regime duration | Predicted probability of regime failures | Actual regime duration | Magnitudes of armed conflict | Regime interruptions by coups |
| --- | --- | --- | --- | --- | --- |
| Predicted regime duration | 1 |  |  |  |  |
| Predicted probability of regime failures | -0.695^***^ | 1 |  |  |  |
| Actual regime duration | 0.374^***^ | -0.269^***^ | 1 |  |  |
| Magnitudes of armed conflict | -0.276^***^ | 0.305^***^ | -0.139^***^ | 1 |  |
| Regime interruptions by coups | -0.299^***^ | 0.482^***^ | -0.203^***^ | 0.075^***^ | 1 |

*Note*: ^*^ *p* < 0.05, ^**^ *p* < 0.01, ^***^ *p* < 0.001.

Figure 2 reveals a positive correlation between authoritarian time horizons and the Corruption Perception Index. An increase in the natural log form of survival time by 1 resulted in a 0.82 increase in the index (0.82, 95% CI = 0.35–1.29) while an increase in **the predicted probability of regime failures by** 0**.1** was associated with a decrease in the index (-1.44, 95% CI = -2.54–-0.33), confirming that shorter-lived regimes were more corrupt. In sum, the evidence from the real-world cases, convergent validation with existing measures and its association with theoretically anticipated outcomes indicate the reliability of our proxy for autocratic time horizons.

In conclusion, following Adcock and Collier [9], we confirmed the validity of a measure of time horizons subjecting it to convergent validation, comparing with the real-world cases and exploring its performance in relation to existing theories that corruption rises as regime horizons shrink.


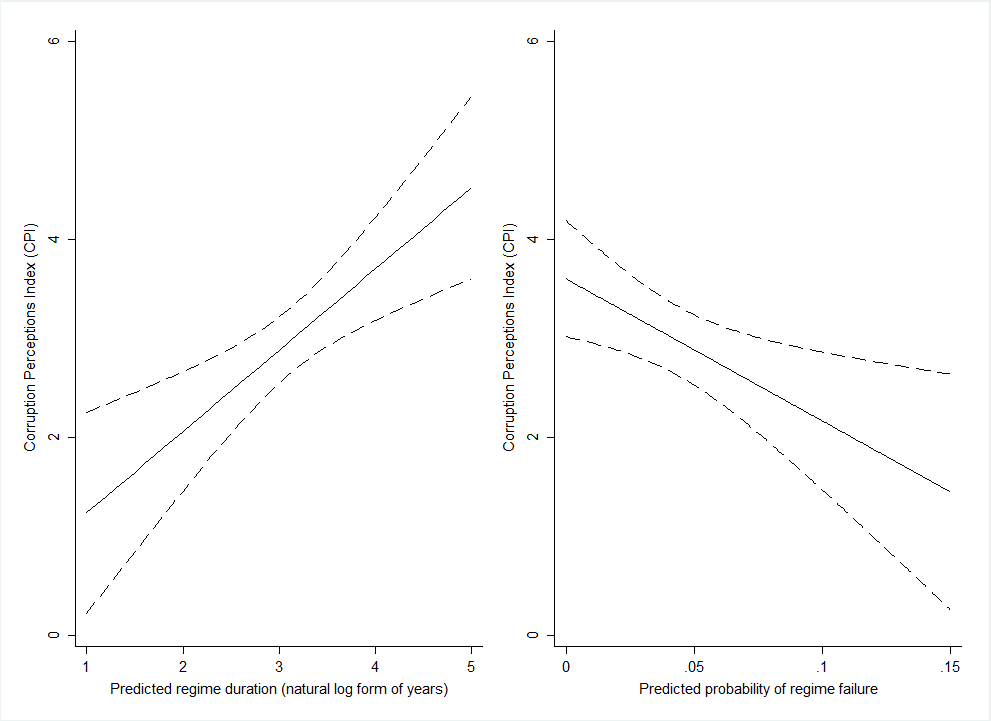


**Figure 2.** Authoritarian Time Horizon and the Level of Corruption, 2008

*Note*: regression coefficient: left panel: 0.821^***^, 95% CI = [0.352, 1.290]; right panel: -14.369^**^, 95% CI = [–25.432, -3.306]; ^*^ *p* < 0.10, ^**^ *p* < 0.05, and ^***^ *p* < 0.01. All results were based on OLS analysis with a list of covariates. *Source*: the author.

**References**

1. Gandhi J, Przeworski A. Cooperation, cooptation and rebellion under dictatorships. Econ Polit. 2006;18:1–26.

2. Chang ECC, Golden MA. Sources of corruption in authoritarian regimes. Soc Sci Quart. 2010;91:1–20.

3. Clague C, Keefer P, Knack S, Olson M. Property and contract rights in autocracies and democracies. J Econ Growth. 1996;1:243–76.

4. Wright J. To invest or insure? How authoritarian time horizons impact foreign aid effectiveness. Comp Polit Stud. 2008;41:971–1000.

5. Ortega B, Casquero A, Sanjuán J. Corruption and convergence in human development: evidence from 69 countries during 1990–2012. Soc Indic Res. 2016;127:691–719.

6. Gerring J, Thacker SC. Political institutions and corruption: the role of unitarism and parliamentarism. Brit J Polit Sci. 2004;34:295–330.

7. Treisman D. The causes of corruption: a cross-national study. J Public Econ. 2000;76:399–457.

8. Esarey J, Schwindt-Bayer LA. Women’s representation, accountability and corruption in democracies. Brit J Polit Sci. 2018;48:659–90.

9. Adcock R, Collier D. Measurement validity: a shared standard for qualitative and quantitative research. Am Polit Sci Rev. 2001;95:529–46.
