# Supplementary material for: How time horizons of autocrats impact health expenditure: a mixed methods research
Source: BMC Public Health. 2020 May 11;20:649. doi: 10.1186/s12889-020-08821-3 (PMC7216651; doi:10.1186/s12889-020-08821-3)
Supplement: Supplementary file 2 — Additional file 2. Supplementary Table 1. The Summary of Variables, Operationalisation of Indicators, Data Sources and Descriptive Statistics. Supplementary Table 2. Authoritarian Regime Duration and Failure, 1945-2010. Supplementary Table 3. Authoritarian Time Horizon and Health Expenditure, 1995-2010. Supplementary Table 4. Health Expenditure Predictor Means. Supplementary Table 5. Country Weights in the Synthetic Country. Supplementary Table 6. Health Expenditure Predictor Means. Supplementary Table 7. Country Weights in the Synthetic Country. Supplementary Table 8. Health Expenditure Predictor Means. Supplementary Table 9. Country Weights in the Synthetic Country. Supplementary Table 10. Health Expenditure and Predictor Means. Supplementary Table 11. Country Weights in the Synthetic Country. Supplementary Table 12. Authoritarian Time Horizon and Health Expenditure, 1995-2010. Supplementary Table 13. Authoritarian Time Horizon and Health Expenditure, 1995-2010 (stable authoritarianism). [file 12889_2020_8821_MOESM2_ESM.docx]

**Supplementary Tables**

**Supplementary Table 1.** The Summary of Variables, Operationalisation of Indicators, Data Sources and Descriptive Statistics

| Variable | Operationalizaiton of indicators | Data Sources |
| --- | --- | --- |
| 1. **Main models: 1995-2010** | | |
| Health expenditure (% of GDP) | The total country spending on health as a proportion of GDP  Continuous variables | The World Bank:  http://data.worldbank.org |
| Predicted regime duration | Continuous variables | The author |
| Multiparty elections | Dummy variables (0: No, 1: Yes) | Database of Political Institutions |
| Prevalence of HIV (% of population ages 15–49) | The percentage of people ages 15–49 who are infected with HIV  Continuous variables | The World Bank:  http://data.worldbank.org |
| GDP per capita growth | Percentage of GDP growth (%)  Continuous variables |  |
| The percentage of other democracies in the world | Percentage of other democracies in the world (%)  Continuous variables | Polity IV Project  https://www.systemicpeace.org/polity/polity4.htm |
| Low-income countries | GNI per capita, calculated using the Atlas method. The thresholds to distinguish between the income groups have evolved over time  Dummy variables (0: No, 1: Yes) | Classifying countries by income, the World Bank:  https://datatopics.worldbank.org/world-development-indicators/stories/the-classification-of-countries-by-income.html |
| Lower-middle income countries | See above  Dummy variables (0: No, 1: Yes) |  |
| Upper-middle income countries | See above  Dummy variables (0: No, 1: Yes) |  |
| High income countries | See above  Dummy variables (0: No, 1: Yes) |  |
| 1. **Generating predicted regime duration: 1945-2010** | | |
| Magnitudes of armed conflict | The systematic and sustained use of lethal violence by organized groups that result in at least 500 directly-related deaths over the course of the episode  Ordinal variables | Major Episodes of Political Violence, 1946–2017  http://www.systemicpeace.org/warlist.htm |
| Ethnic fractionalization | $1-\sum_{i=1}^{n} s_{\mathrm{ij}}^{2}$, where s_ij_ is the share of ethnic group i (i = 1...n) in country j  Continuous variables (0-1) | Fearon, J.D., 2003. Ethnic and cultural diversity by country. J. Econ. Growth 8(2), 195–222 |
| Religious fractionalization | $1-\sum_{i=1}^{n} s_{\mathrm{ij}}^{2}$, where s_ij_ is the share of religious group i (i = 1...n) in country j  Continuous variables (0-1) | Alesina, A., Devleeschauwer, A., Easterly, W., Kurlat, S., Wacziarg, R., 2003. Fractionalization. J. Econ. Growth 8(2), 155–194 |
| GDP per capita | Real GDP per capita in 2011US$, 2011 benchmark  Continuous variables | Maddison Project Database 2018  https://www.rug.nl/ggdc/historicaldevelopment/maddison/releases/maddison-project-database-2018 |
| Military regimes | Control over policy, leadership selection, and the security apparatus is in the hands of the military  Dummy variables (0: No, 1: Yes) | Autocratic Regime Data  http://sites.psu.edu/dictators |
| Monarchy | Control over policy, leadership selection, and the security apparatus is in the hands of a royal family  Dummy variables (0: No, 1: Yes) |  |
| Oligarchy | Leaders are chosen through competitive elections but most of the population is disenfranchised  Dummy variables (0: No, 1: Yes) |  |
| Party regimes | Control over policy, leadership selection, and the security apparatus is in the hands of a ruling party  Dummy variables (0: No, 1: Yes) |  |
| Personalist regimes | Control over policy, leadership selection, and the security apparatus is in the hands of a narrower group centered on an individual dictator  Dummy variables (0: No, 1: Yes) |  |
| Military-personal hybrid | Hybrids of military and personalist regimes  Dummy variables (0: No, 1: Yes) |  |
| Party-military hybrid | Hybrids of party and military regimes  Dummy variables (0: No, 1: Yes) |  |
| Party-personal hybrid | Hybrids of party and personalist regimes  Dummy variables (0: No, 1: Yes) |  |
| Party-military-personalist hybrid | Hybrids of party, military and personalist regimes  Dummy variables (0: No, 1: Yes) |  |
| 1. **Other measures of autocratic time horizon** | | |
| Predicted probability of regime failure | Continuous variables | The author |
| Actual regime duration | Consecutive years in which the same autocratic regime has been in power in a particular country up to time *t*  Continuous variables | Autocratic Regime Data  http://sites.psu.edu/dictators |
| Regime interruptions by coups | An authoritarian regime follows the last year of the regime overthrown by military coup (defined as ouster by the military of the regime in power)  Dummy variables (0: No, 1: Yes) | Autocratic Regimes Code Book  http://sites.psu.edu/dictators |
| 1. **Corruption: 2008** | | |
| Level of corruption | The abuse of public power for private gain  Continuous variables (Corruption Perception Index, 1–10: most to least corrupt) | Transparency International  https://www.transparency.org/research/cpi/overview |
| Executive constraints* | The extent of institutionalised constraints on the decision making powers of chief executives  Ordinal variables | Polity IV Project  https://www.systemicpeace.org/polity/polity4.htm |
| British colonies* | A country that had been ruled by the British Empire  Dummy variables (0: No, 1: Yes) | Replication data for Fearon, J.D., Laitin, D.D., 2003. Ethnicity, insurgency, and civil war. Am. Pol. Sci. Rev. 97(1), 75–90 |
| Federal system* | A constitutional design that stipulates a system of government in which power is divided between a central authority and constituent political units  Dummy variables (0: No, 1: Yes) | SBS World Guide 16^th^ Edition |
| Women in national parliaments* | Proportion of seats held by women in national parliaments (%)  Continuous variables | Inter-Parliamentary Union  http://archive.ipu.org/wmn-e/classif-arc.htm |

| Variable | Spell | N | Mean | S.D. | Min | Max |
| --- | --- | --- | --- | --- | --- | --- |
| Health expenditure (% of GDP) | 1995–2010 | 1159 | 4.843 | 1.806 | 1.446 | 13.633 |
| Predicted regime duration | 1945–2010 | 4296 | 25.250 | 17.491 | 2.097 | 89.095 |
| Log(predicted regime duration) | 1945–2010 | 4296 | 2.970 | 0.761 | 0.740 | 4.490 |
| Multiparty elections | 1995–2010 | 1208 | 0.695 | 0.460 | 0 | 1 |
| Prevalence of HIV (% of population ages 15–49) | 1995–2010 | 1192 | 2.345 | 4.616 | 0 | 30 |
| GDP per capita growth (%) | 1995–2010 | 1137 | 3.038 | 7.847 | -31.342 | 140.501 |
| The percentage of other democracies in the world | 1995–2010 | 1208 | 53.408 | 4.181 | 47.853 | 59.036 |
| Low-income countries | 1995–2010 | 1115 | 0.536 | 0.499 | 0 | 1 |
| Lower-middle income countries | 1995–2010 | 1115 | 0.283 | 0.450 | 0 | 1 |
| Upper-middle income countries | 1995–2010 | 1115 | 0.107 | 0.309 | 0 | 1 |
| High income countries | 1995–2010 | 1115 | 0.074 | 0.263 | 0 | 1 |
| Magnitudes of armed conflict | 1945–2010 | 4581 | 0.933 | 2.079 | 0 | 18 |
| Ethnic fractionalization | 1945–2010 | 4582 | 0.502 | 0.273 | 0.002 | 0.953 |
| Religious fractionalization | 1945–2010 | 4545 | 0.414 | 0.242 | 0.000 | 0.860 |
| GDP per capita | 1945–2010 | 4353 | 6929.669 | 12324.69 | 280 | 141662 |
| Log(GDP per capita) | 1945–2010 | 4353 | 8.178 | 1.065 | 5.635 | 11.861 |
| Military regimes | 1945–2010 | 4591 | 0.071 | 0.257 | 0 | 1 |
| Monarchy | 1945–2010 | 4591 | 0.130 | 0.336 | 0 | 1 |
| Oligarchy | 1945–2010 | 4591 | 0.014 | 0.119 | 0 | 1 |
| Party regimes | 1945–2010 | 4591 | 0.325 | 0.469 | 0 | 1 |
| Personalist regimes | 1945–2010 | 4591 | 0.251 | 0.434 | 0 | 1 |
| Military-personal hybrid | 1945–2010 | 4591 | 0.055 | 0.229 | 0 | 1 |
| Party-military hybrid | 1945–2010 | 4591 | 0.029 | 0.168 | 0 | 1 |
| Party-personal hybrid | 1945–2010 | 4591 | 0.086 | 0.280 | 0 | 1 |
| Party-military-personalist hybrid | 1945–2010 | 4591 | 0.039 | 0.193 | 0 | 1 |
| Predicted probability of regime failure | 1945–2010 | 4296 | 0.050 | 0.044 | ~0 | 0.317 |
| Actual regime duration | 1945–2010 | 4591 | 22.019 | 30.815 | 1 | 269 |
| Regime interruptions by coups | 1945–2010 | 4591 | 0.293 | 0.455 | 0 | 1 |
| Level of corruption | 2008 | 56 | 2.855 | 1.297 | 1.4 | 9.2 |
| Executive constraints* | 2008 | 55 | 2.691 | 1.052 | 1 | 5 |
| British colonies* | 2008 | 57 | 0.298 | 0.462 | 0 | 1 |
| Federal system* | 2008 | 57 | 0.123 | 0.331 | 0 | 1 |
| Women in national parliaments* | 2008 | 54 | 16.572 | 11.228 | 0 | 56.3 |

*Note*: *A list of control variables based on the comparative literature on corruption, including institutional factors, histories of British rule and women’s representation in the parliament, was incorporated into our analyses of the level of corruption in authoritarian polities. *Source*: the author.

**Supplementary Table 2.** Authoritarian Regime Duration and Failure, 1945–2010

|  | Model (1)  duration | | Model (2)  duration | | Model (3)  failure | |
| --- | --- | --- | --- | --- | --- | --- |
|  | Coef. | HR | Coef. | HR | Coef. | OR |
| Military regime | 1.002^***^ | 2.725^***^ | 1.137^***^ | 3.116^***^ | 1.079^***^ | 2.941^***^ |
|  | (0.189) | (0.515) | (0.197) | (0.615) | (0.211) | (0.621) |
| Military– personal regime | 0.156 | 1.169 | 0.173 | 1.188 | 0.175 | 1.191 |
|  | (0.243) | (0.284) | (0.244) | (0.290) | (0.256) | (0.305) |
| Monarchy | -0.920^***^ | 0.399^***^ | -1.121^***^ | 0.326^***^ | -1.263^***^ | 0.283^***^ |
|  | (0.322) | (0.128) | (0.331) | (0.108) | (0.368) | (0.104) |
| Oligarchy | -0.347 | 0.707 | -0.472 | 0.624 | -0.598 | 0.550 |
|  | (0.604) | (0.427) | (0.608) | (0.380) | (0.675) | (0.371) |
| Party regime | -0.948^***^ | 0.387^***^ | -1.042^***^ | 0.353^***^ | -1.079^***^ | 0.340^***^ |
|  | (0.214) | (0.083) | (0.217) | (0.076) | (0.229) | (0.078) |
| Party– military regime | -0.189 | 0.828 | -0.211 | 0.809 | -0.219 | 0.803 |
|  | (0.429) | (0.356) | (0.430) | (0.348) | (0.442) | (0.355) |
| Party– military– personal | -1.727^**^ | 0.178^**^ | -1.841^**^ | 0.159^**^ | -1.884^**^ | 0.152^**^ |
|  | (0.720) | (0.128) | (0.722) | (0.114) | (0.728) | (0.111) |
| Party– personal regime | -0.703^**^ | 0.495^**^ | -0.766^**^ | 0.465^**^ | -0.782^**^ | 0.457^**^ |
|  | (0.340) | (0.168) | (0.341) | (0.159) | (0.349) | (0.160) |
| Personal regime (ref. ) |  |  |  |  |  |  |
|  |  |  |  |  |  |  |
| Log (GDP pc ) | -0.215^***^ | 0.806^***^ | -0.258^***^ | 0.772^***^ | -0.248^***^ | 0.780^***^ |
|  | (0.079) | (0.064) | (0.082) | (0.063) | (0.084) | (0.066) |
| Ethnic fractionalization | 0.371 | 1.449 | 0.402 | 1.495 | 0.296 | 1.344 |
|  | (0.300) | (0.435) | (0.301) | (0.450) | (0.313) | (0.420) |
| Religious fractionalization | -0.777^**^ | 0.460^**^ | -0.837^**^ | 0.433^**^ | -0.890^**^ | 0.411^**^ |
|  | (0.335) | (0.154) | (0.335) | (0.145) | (0.351) | (0.144) |
| Armed conflict | 0.070^**^ | 1.072^**^ | 0.073^**^ | 1.076^**^ | 0.072^**^ | 1.074^**^ |
|  | (0.029) | (0.031) | (0.030) | (0.032) | (0.031) | (0.033) |
| No. of observations | 4296 | | 4296 | | 4296 | |
| No. of Subjects | 270 | | 270 | | 270 | |
| LR chi^2^ | 130.75 | | 127.79 | | 146.81 | |
| Prob> chi^2^ | 0.0000 | | 0.0000 | | 0.0005 | |
| Log likelihood | -352.93507 | | -349.56927 | | -777.05941 | |

*Note*: standard error in parentheses in parentheses, ^*^ *p* < 0.1, ^**^ *p* < 0.05, ^***^ *p* < 0.01. Model 1: this study used parametric survival model in which survival time is assumed to follow exponential distribution. Model 2: this study changed parametric survival model in which survival time is assumed to follow Weibull distribution. Model 3: this study used logistic regression with a cubic polynomial of time.

**Supplementary Table 3.** Authoritarian Time Horizon and Health Expenditure, 1995–2010

|  | Model (1)  HE | Model (2)  HE | Model (3)  HE | Model (4)  HE |
| --- | --- | --- | --- | --- |
| Lag. predicted regime duration | 1.143^***^ | 1.576^***^ |  |  |
|  | (0.277) | (0.328) |  |  |
| Lag. actual regime duration |  |  | 0.027^***^ |  |
|  |  |  | (0.008) |  |
| Lag. predicted regime failure |  |  |  | -11.206^***^ |
|  |  |  |  | (3.331) |
| Elections | 0.112 | 0.113 | 0.115 | 0.104 |
|  | (0.131) | (0.148) | (0.131) | (0.132) |
| Prevalence of HIV | -0.054 | -0.178^***^ | -0.032 | -0.033 |
|  | (0.043) | (0.059) | (0.043) | (0.043) |
| Constant | 1.156 | 0.008 | 3.867^***^ | 5.054^***^ |
|  | (0.816) | (0.976) | (0.243) | (0.257) |
| No. of subjects | 887 | 717 | 903 | 887 |
| No. of groups | 72 | 61 | 73 | 72 |
| Years dummy | Yes | Yes | Yes | Yes |
| Adjusted R-squared | 0.7388 | 0.7471 | 0.7357 | 0.7370 |
| Prob>F | 0.0000 | 0.0000 | 0.0000 | 0.0000 |

*Note*: standard error in parentheses; ^*^ *p* < 0.1, ^**^ *p* < 0.05, and ^***^ *p* < 0.01. Independent variables are lagged by one year. Model 1: Hausman test: Prob. >chi2 = 0.000, and a joint test of year dummies: Prob. > F = 0.000; model 2: Hausman test: Prob. >chi2 = 0.000, and a joint test of year dummies: Prob. > F = 0.028; model 3: Hausman test: Prob. >chi2 = 0.000, and a joint test of year dummies: Prob. > F = 0.062; model 4: Hausman test: Prob. >chi2 = 0.014, and a joint test of year dummies: Prob. > F = 0.000. Model 1: this study defined dictatorships as those with Polity IV scores lower than 6; model 2: this study changed the threshold for the determinant of dictatorships as those with Polity IV scores lower than 1; model 3: this study used the actual regime duration as a proxy of time horizons; model 4: this study measured authoritarian time horizon using the predicted probability of regime failures.

**Supplementary Table 4.** Health Expenditure Predictor Means

|  | Chad | | Rwanda | | Ivory Coast | |
| --- | --- | --- | --- | --- | --- | --- |
| Predictors | Real | Synthetic | Real | Synthetic | Real | Synthetic |
| Log(GDP per capita) | 7.023 | 7.030 | 6.681 | 6.608 | 8.029 | 8.020 |
| Multiparty elections | 0.778 | 0.782 | 0 | 0.361 | 1 | 0.999 |
| Lagged health expenditure | 6.024 | 6.025 | 4.482 | 4.433 | 6.868 | 6.865 |

*Note*: All variables were averaged for the entire pre-intervention period (e.g. Chad: 1996–2004; Rwanda: 1996–2002; Ivory Coast: 1996–1999).

**Supplementary Table 5.** Country Weights in the Synthetic Country

| Chad | | Rwanda | | Ivory Coast | |
| --- | --- | --- | --- | --- | --- |
| Country | Weights | Country | Weights | Country | Weights |
| Armenia | 0 | Algeria | 0 | Armenia | 0.156 |
| Bahrain | 0 | Angola | 0 | Bahrain | 0 |
| Belarus | 0 | Burundi | 0 | Belarus | 0 |
| Bhutan | 0.218 | Congo Kinshasa | 0.579 | Bhutan | 0 |
| Burkina Faso | 0 | Myanmar | 0 | Burkina Faso | 0.010 |
| Cameroon | 0 | Nigeria | 0 | Cameroon | 0.045 |
| Djibouti | 0 | Sudan | 0 | Djibouti | 0.011 |
| Equatorial Guinea | 0 | Uganda | 0.421 | Equatorial Guinea | 0.007 |
| Gabon | 0 |  |  | Gabon | 0.005 |
| Gambia | 0 |  |  | Gambia | 0 |
| Iran | 0 |  |  | Iran | 0.006 |
| Jordan | 0.106 |  |  | Jordan | 0.442 |
| Kazakhstan | 0 |  |  | Kazakhstan | 0.003 |
| Kuwait | 0 |  |  | Kuwait | 0 |
| Laos | 0 |  |  | Laos | 0 |
| Mauritania | 0 |  |  | Mauritania | 0.008 |
| Morocco | 0 |  |  | Morocco | 0.009 |
| Mozambique | 0.676 |  |  | Mozambique | 0.195 |
| Oman | 0 |  |  | Oman | 0 |
| Singapore | 0 |  |  | Singapore | 0.004 |
| Suriname | 0 |  |  | Suriname | 0.043 |
| Swaziland | 0 |  |  | Swaziland | 0 |
| Tanzania | 0 |  |  | Tanzania | 0.016 |
| Togo | 0 |  |  | Togo | 0.020 |
| Tunisia | 0 |  |  | Tunisia | 0.011 |
| Turkmenistan | 0 |  |  | Turkmenistan | 0.008 |
| UAE | 0 |  |  | UAE | 0 |
| Uzbekistan | 0 |  |  | Uzbekistan | 0 |
| Vietnam | 0 |  |  | Vietnam | 0 |

*Note*: for a synthetic version of Chad and Ivory Coast, we chose countries with no armed conflict for the entire pre-intervention period. For a synthetic version of Rwanda, we chose countries where armed conflict exists for the entire pre-intervention period.

**Supplementary Table 6.** Health Expenditure Predictor Means

|  | Chad | | Rwanda | | Ivory Coast | |
| --- | --- | --- | --- | --- | --- | --- |
| Predictors | Real | Synthetic | Real | Synthetic | Real | Synthetic |
| Log(GDP per capita) | 7.023 | 7.483 | 6.681 | 6.660 | 8.029 | 8.851 |
| Multiparty elections | 0.778 | 0.888 | 0 | 0.368 | 1 | 0.876 |
| Lagged health expenditure | 6.024 | 6.022 | 4.482 | 4.445 | 6.868 | 6.858 |
| Prevalence of HIV | 1.911 | 1.766 | 5.157 | 5.286 | 6.300 | 0.509 |
| Constitutional design | 0 | 0 | 0 | 0 | 1 | 0.972 |
| Dominant party regimes | 0 | 0.033 | 0 | 0.002 | 1 | 0.576 |

*Note*: All variables were averaged for the entire pre-intervention period (e.g. Chad: 1996–2004; Rwanda: 1996–2002; Ivory Coast: 1996–1999).

**Supplementary Table 7.** Country Weights in the Synthetic Country

| Chad | | Rwanda | | Ivory Coast | |
| --- | --- | --- | --- | --- | --- |
| Country | Weights | Country | Weights | Country | Weights |
| Armenia | 0 | Algeria | 0 | Armenia | 0 |
| Bahrain | 0 | Angola | 0.002 | Bahrain | 0 |
| Belarus | 0 | Burundi | 0 | Belarus | 0 |
| Bhutan | 0 | Congo Kinshasa | 0.544 | Bhutan | 0 |
| Burkina Faso | 0.558 | Myanmar | 0 | Burkina Faso | 0 |
| Cameroon | 0 | Nigeria | 0 | Cameroon | 0 |
| Djibouti | 0 | Sudan | 0.038 | Djibouti | 0 |
| Equatorial Guinea | 0 | Uganda | 0.416 | Equatorial Guinea | 0 |
| Gabon | 0 |  |  | Gabon | 0 |
| Gambia | 0 |  |  | Gambia | 0 |
| Iran | 0 |  |  | Iran | 0 |
| Jordan | 0.158 |  |  | Jordan | 0 |
| Kazakhstan | 0 |  |  | Kazakhstan | 0 |
| Kuwait | 0 |  |  | Kuwait | 0 |
| Laos | 0 |  |  | Laos | 0 |
| Mauritania | 0 |  |  | Mauritania | 0 |
| Morocco | 0 |  |  | Morocco | 0 |
| Mozambique | 0.033 |  |  | Mozambique | 0 |
| Oman | 0 |  |  | Oman | 0 |
| Singapore | 0 |  |  | Singapore | 0 |
| Suriname | 0 |  |  | Suriname | 0.424 |
| Swaziland | 0 |  |  | Swaziland | 0 |
| Tanzania | 0 |  |  | Tanzania | 0 |
| Togo | 0 |  |  | Togo | 0 |
| Tunisia | 0 |  |  | Tunisia | 0.452 |
| Turkmenistan | 0 |  |  | Turkmenistan | 0 |
| UAE | 0 |  |  | UAE | 0 |
| Uzbekistan | 0.251 |  |  | Uzbekistan | 0 |
| Vietnam | 0 |  |  | Vietnam | 0.124 |

*Note*: for a synthetic version of Chad and Ivory Coast, we chose countries with no armed conflict for the entire pre-intervention period. For a synthetic version of Rwanda, we chose countries where armed conflict exists for the entire pre-intervention period.

**Supplementary Table 8.** Health Expenditure Predictor Means

|  | Chad | | | | |
| --- | --- | --- | --- | --- | --- |
| Predictors | Real | Synthetic I | Synthetic II | Synthetic III | Synthetic IV |
| Log(GDP per capita) | 7.023 | 7.483 | 7.077 | 7.102 | 7.464 |
| Multiparty elections | 0.778 | 0.888 | 0.828 | 0.823 | 0.871 |
| Lagged health expenditure | 6.024 | 6.022 | 6.023 | 6.022 | 6.015 |

*Note*: All variables were averaged for the entire pre-intervention period. We constructed a synthetic Chad using all countries in the donor pool (Synthetic I), omitting Bhutan (Synthetic II), Jordan (Synthetic III) and Mozambique (Synthetic IV).

**Supplementary Table 9.** Country Weights in the Synthetic Country

| Chad | | | | |
| --- | --- | --- | --- | --- |
| Country | Weights I | Weights II | Weights III | Weights IV |
| Armenia | 0 | 0 | 0 | 0 |
| Bahrain | 0 | 0 | 0 | 0 |
| Belarus | 0 | 0 | 0 | 0 |
| Bhutan | 0.218 | Excluding | 0.177 | 0.128 |
| Burkina Faso | 0 | 0 | 0 | 0.700 |
| Cameroon | 0 | 0 | 0 | 0 |
| Djibouti | 0 | 0 | 0 | 0 |
| Equatorial Guinea | 0 | 0 | 0 | 0 |
| Gabon | 0 | 0 | 0 | 0 |
| Gambia | 0 | 0 | 0 | 0 |
| Iran | 0 | 0 | 0 | 0 |
| Jordan | 0.106 | 0.180 | Excluding | 0.171 |
| Kazakhstan | 0 | 0 | 0 | 0 |
| Kuwait | 0 | 0 | 0 | 0 |
| Laos | 0 | 0 | 0 | 0 |
| Mauritania | 0 | 0 | 0 | 0 |
| Morocco | 0 | 0 | 0 | 0 |
| Mozambique | 0.676 | 0.648 | 0.678 | Excluding |
| Oman | 0 | 0 | 0 | 0 |
| Singapore | 0 | 0 | 0 | 0 |
| Suriname | 0 | 0 | 0.145 | 0 |
| Swaziland | 0 | 0 | 0 | 0 |
| Tanzania | 0 | 0 | 0 | 0 |
| Togo | 0 | 0 | 0 | 0 |
| Tunisia | 0 | 0 | 0 | 0 |
| Turkmenistan | 0 | 0 | 0 | 0 |
| UAE | 0 | 0 | 0 | 0 |
| Uzbekistan | 0 | 0 | 0 | 0 |
| Vietnam | 0 | 0.172 | 0 | 0 |

*Note*: for a synthetic version of Chad, we chose countries with no armed conflict for the entire pre-intervention period, and use all countries in the donor pool (weights I), excluding Bhutan (weights II), Jordan (weights III) and Mozambique (weights IV) that that received a positive weight in the second column.

**Supplementary Table 10.** Health Expenditure Predictor Means

|  | Chad | | Rwanda | | Ivory Coast | |
| --- | --- | --- | --- | --- | --- | --- |
| Predictors | Real | Synthetic | Real | Synthetic | Real | Synthetic |
| Log(GDP per capita) | 6.979 | 7.071 | 6.660 | 6.817 | 8.027 | 8.034 |
| Multiparty elections | 0.750 | 0.786 | 0 | 0.356 | 1 | 1 |
| Lagged health expenditure | 6.091 | 6.090 | 4.499 | 4.451 | 6.725 | 6.730 |

*Note*: All variables were averaged for the assumed pre-intervention period (e.g. Chad: 1996–2003; Rwanda: 1996–2001; Ivory Coast: 1996–1998).

**Supplementary Table 11.** Country Weights in the Synthetic Country

| Chad | | Rwanda | | Ivory Coast | |
| --- | --- | --- | --- | --- | --- |
| Country | Weights | Country | Weights | Country | Weights |
| Armenia | 0 | Algeria | 0 | Armenia | 0.046 |
| Bahrain | 0 | Angola | 0 | Bahrain | 0 |
| Belarus | 0 | Burundi | 0 | Belarus | 0 |
| Bhutan | 0.214 | Congo Kinshasa | 0.441 | Bhutan | 0 |
| Burkina Faso | 0 | Myanmar | 0 | Burkina Faso | 0.030 |
| Cameroon | 0 | Nigeria | 0 | Cameroon | 0.010 |
| Djibouti | 0 | Sudan | 0.165 | Djibouti | 0.011 |
| Equatorial Guinea | 0 | Uganda | 0.394 | Equatorial Guinea | 0.005 |
| Gabon | 0 |  |  | Gabon | 0.002 |
| Gambia | 0 |  |  | Gambia | 0 |
| Iran | 0 |  |  | Iran | 0.004 |
| Jordan | 0.134 |  |  | Jordan | 0.470 |
| Kazakhstan | 0 |  |  | Kazakhstan | 0.005 |
| Kuwait | 0 |  |  | Kuwait | 0 |
| Laos | 0 |  |  | Laos | 0 |
| Mauritania | 0 |  |  | Mauritania | 0.003 |
| Morocco | 0 |  |  | Morocco | 0.008 |
| Mozambique | 0.652 |  |  | Mozambique | 0.159 |
| Oman | 0 |  |  | Oman | 0 |
| Singapore | 0 |  |  | Singapore | 0.002 |
| Suriname | 0 |  |  | Suriname | 0.056 |
| Swaziland | 0 |  |  | Swaziland | 0 |
| Tanzania | 0 |  |  | Tanzania | 0.004 |
| Togo | 0 |  |  | Togo | 0.171 |
| Tunisia | 0 |  |  | Tunisia | 0.010 |
| Turkmenistan | 0 |  |  | Turkmenistan | 0.005 |
| UAE | 0 |  |  | UAE | 0 |
| Uzbekistan | 0 |  |  | Uzbekistan | 0 |
| Vietnam | 0 |  |  | Vietnam | 0 |

*Note*: for a synthetic version of Chad and Ivory Coast, we chose countries with no armed conflict for the assumed pre-intervention period. For a synthetic version of Rwanda, we chose countries where armed conflict exists for the assumed pre-intervention period.

**Supplementary Table 12.** Authoritarian Time Horizon and Health Expenditure, 1995–2010

|  | Model (1)  HE | Model (2)  HE | Model (3)  HE | Model (4)  HE |
| --- | --- | --- | --- | --- |
| Lag. predicted regime duration | 1.129^***^ | 1.585^***^ |  |  |
|  | (0.279) | (0.330) |  |  |
| Lag. actual regime duration |  |  | 0.028^***^ |  |
|  |  |  | (0.008) |  |
| Lag. predicted regime failure |  |  |  | -10.949^***^ |
|  |  |  |  | (3.352) |
| GDP per capita growth | -0.004 | -0.008 | -0.002 | -0.003 |
|  | (0.006) | (0.007) | (0.006) | (0.006) |
| Elections | 0.111 | 0.109 | 0.108 | 0.102 |
|  | (0.131) | (0.147) | (0.132) | (0.132) |
| Prevalence of HIV | -0.052 | -0.164^***^ | -0.029 | -0.030 |
|  | (0.043) | (0.059) | (0.043) | (0.043) |
| Constant | 1.253 | -0.015 | 3.879^***^ | 5.078^***^ |
|  | (0.815) | (0.978) | (0.246) | (0.265) |
| No. of subjects | 867 | 709 | 883 | 867 |
| No. of groups | 71 | 61 | 72 | 71 |
| Years dummy | Yes | Yes | Yes | Yes |
| Adjusted R-squared | 0.7387 | 0.7503 | 0.7359 | 0.7368 |
| Prob>F | 0.0000 | 0.0000 | 0.0000 | 0.0000 |

*Note*: standard error in parentheses; ^*^ *p* < 0.1, ^**^ *p* < 0.05, and ^***^ *p* < 0.01. Independent variables are lagged by one year. Model 1: Hausman test: Prob. >chi2 = 0.000, and a joint test of year dummies: Prob. > F = 0.000; model 2: Hausman test: Prob. >chi2 = 0.000, and a joint test of year dummies: Prob. > F = 0.011; model 3: Hausman test: Prob. >chi2 = 0.000, and a joint test of year dummies: Prob. > F =0.043; model 4: Hausman test: Prob. >chi2 = 0.015, and a joint test of year dummies: Prob. > F = 0.000. Model 1: this study defined dictatorships as those with Polity IV scores lower than 6; model 2: this study changed the threshold for the determinant of dictatorships as those with Polity IV scores lower than 1; model 3: this study used the actual regime duration as a proxy of time horizons; model 4: this study measured authoritarian time horizon using the predicted probability of regime failures. The data of GDP per capita growth was from the World Bank.

**Supplementary Table 13.** Authoritarian Time Horizon and Health Expenditure, 1995–2010 (stable authoritarianism)

|  | Model (1)  HE | Model (2)  HE | Model (3)  HE | Model (4)  HE |
| --- | --- | --- | --- | --- |
| Lag. predicted regime duration | 1.210^***^ | 1.626^***^ |  |  |
|  | (0.291) | (0.330) |  |  |
| Lag. actual regime duration |  |  | 0.041^***^ |  |
|  |  |  | (0.006) |  |
| Lag. predicted regime failure |  |  |  | -11.581^***^ |
|  |  |  |  | (3.465) |
| Elections | 0.144 | 0.101 | 0.168 | 0.140 |
|  | (0.141) | (0.153) | (0.141) | (0.142) |
| Prevalence of HIV | -0.060 | -0.174^***^ | -0.059 | -0.035 |
|  | (0.046) | (0.061) | (0.044) | (0.045) |
| Constant | 0.839 | -0.202 | 3.570^***^ | 4.969^***^ |
|  | (0.866) | (1.013) | (0.232) | (0.269) |
| No. of subjects | 775 | 676 | 791 | 775 |
| No. of groups | 50 | 49 | 51 | 50 |
| Years dummy | Yes | Yes | No | Yes |
| Adjusted R-squared | 0.7142 | 0.7317 | 0.7080 | 0.7118 |
| Prob>F | 0.0000 | 0.0000 | 0.0000 | 0.0000 |

*Note*: standard error in parentheses; ^*^ *p* < 0.1, ^**^ *p* < 0.05, and ^***^ *p* < 0.01. Independent variables are lagged by one year. Model 1: Hausman test: Prob. >chi2 = 0.000, and a joint test of year dummies: Prob. > F = 0.001; model 2: Hausman test: Prob. >chi2 = 0.000, and a joint test of year dummies: Prob. > F = 0.035; model 3: Hausman test: Prob. >chi2 = 0.000, and a joint test of year dummies: Prob. > F =0.187; model 4: Hausman test: Prob. >chi2 = 0.018, and a joint test of year dummies: Prob. > F = 0.000. Model 1: this study defined dictatorships as those with Polity IV scores lower than 6; model 2: this study changed the threshold for the determinant of dictatorships as those with Polity IV scores lower than 1; model 3: this study used the actual regime duration as a proxy of time horizons; model 4: this study measured authoritarian time horizon using the predicted probability of regime failures. Stable authoritarianism was defined when an authoritarian regime survived the period between 1995 and 2010.
