# Supplementary material for: How time horizons of autocrats impact health expenditure: a mixed methods research
Source: BMC Public Health. 2020 May 11;20:649. doi: 10.1186/s12889-020-08821-3 (PMC7216651; doi:10.1186/s12889-020-08821-3)
Supplement: Supplementary file 3 — Additional file 3. Supplementary Figure 1. Trends in Health Expenditure: More Predictors. Supplementary Figure 2. Trends in Health Expenditure: The Leave-one-out Estimates. Supplementary Figure 3. Placebo Armed Conflict-Trends in Health Expenditure: Actual Country vs. Synthetic Country. Supplementary Figure 4. Ratio of Pre-treatment RMSPE to Post-treatment RMSPE: Chad and Control Countries. Supplementary Figure 5. Health Expenditure Gap: Actual Country versus Synthetic Country. Supplementary Figure 6. Authoritarian Time Horizon and Health Expenditure, 1995-2010. Supplementary Figure 7. Authoritarian Time Horizon and Health Expenditure, 1995-2010. Supplementary Figure 8. Authoritarian Time Horizon and Health Expenditure, 1995-2010. Supplementary Figure 9. Authoritarian Time Horizon and Health Expenditure, 1995-2010. Supplementary Figure 10. IV Analysis: The Coefficient Plot of Predicted Regime Duration, 1995-2010. [file 12889_2020_8821_MOESM3_ESM.docx]

**Supplementary Figures**

**Supplementary Figure 1.** Trends in Health Expenditure: More Predictors

*Note*: health expenditure from 1996 to 2010 (upper panel): actual Chad (5.410, 5.557, 5.412, 5.866, 6.277, 6.086, 8.332, 5.491, 5.720, 3.910, 3.322, 3.346, 2.916, 3.307, 2.949) and synthetic Chad (6.203, 6.142, 6.138, 5.982, 5.881, 5.783, 6.043, 6.085, 6.510, 6.748, 6.542, 6.650, 6.812, 7.404, 6.850); health expenditure from 1996 to 2004 (middle panel): actual Rwanda (4.435, 4.417, 5.078, 4.573, 4.221, 4.380, 4.176, 6.342, 6.245) and synthetic Rwanda (4.184, 4.451, 5.062, 4.791, 3.733, 4.595, 4.764, 5.008, 5.090); health expenditure from 1996 to 2010 (lower panel): actual Ivory Coast (6.741, 7.066, 7.298, 6.522, 6.001, 4.846, 4.467, 4.652, 5.237, 5.390, 5.871, 6.346, 6.206, 6.414, 6.324) and synthetic Ivory Coast (6.224, 7.192, 7.768, 6.836, 7.137, 6.585, 6.015, 5.830, 6.089, 6.056, 5.877, 5.817, 5.741, 6.146, 6.208). Supplementary Table 6 compares the characteristics of the three countries of interest prior to the intervention to those of the individual synthetic version. Supplementary Table 7 shows the weight of each country in the synthetic control. *Source*: the author.

**Supplementary Figure 2.** Trends in Health Expenditure: The Leave-one-out Estimates

*Note*: health expenditure from 1996 to 2010 (upper panel): actual Chad (5.410, 5.557, 5.412, 5.866, 6.277, 6.086, 8.332, 5.491, 5.720, 3.910, 3.322, 3.346, 2.916, 3.307, 2.949), synthetic Chad (6.203, 6.142, 6.138, 5.982, 5.881, 5.783, 6.043, 6.085, 6.510, 6.748, 6.542, 6.650, 6.812, 7.404, 6.850), synthetic Chad (excluding Bhutan, 6.203, 6.142, 6.138, 5.982, 5.881, 5.783, 6.043, 6.085, 6.510, 6.748, 6.542, 6.650, 6.812, 7.404, 6.850), synthetic Chad (excluding Jordan, 6.203, 6.142, 6.138, 5.982, 5.881, 5.783, 6.043, 6.085, 6.510, 6.748, 6.542, 6.650, 6.812, 7.404, 6.850), synthetic Chad (excluding Mozambique, 6.203, 6.142, 6.138, 5.982, 5.881, 5.783, 6.043, 6.085, 6.510, 6.748, 6.542, 6.650, 6.812, 7.404, 6.850). Supplementary Table 8 compares the characteristics of Chad prior to the intervention to those of four synthetic versions. Supplementary Table 9 shows the weight of Chad in the synthetic controls. *Source*: the author.

**Supplementary Figure 3.** Placebo Armed Conflict-Trends in Health Expenditure: Actual Country vs. Synthetic Country

*Note*: health expenditure from 1996 to 2010 (upper panel): actual Chad (5.410, 5.557, 5.412, 5.866, 6.277, 6.086, 8.332, 5.491, 5.720, 3.910, 3.322, 3.346, 2.916, 3.307, 2.949) and synthetic Chad (5.337, 5.794, 5.743, 6.185, 6.790, 6.271, 7.161, 6.444, 6.011, 6.798, 6.457, 5.801, 5.788, 6.112, 5.743); health expenditure from 1996 to 2004 (middle panel): actual Rwanda (4.435, 4.417, 5.078, 4.573, 4.221, 4.380, 4.176, 6.342, 6.245) and synthetic Rwanda (4.305, 4.486, 5.002, 4.742, 3.839, 4.526, 4.694, 4.906, 5.002); health expenditure from 1996 to 2010 (lower panel): actual Ivory Coast (6.741, 7.066, 7.298, 6.522, 6.001, 4.846, 4.467, 4.652, 5.237, 5.390, 5.871, 6.346, 6.206, 6.414, 6.324) and synthetic Ivory Coast (6.676, 6.890, 7.126, 7.153, 7.546, 7.470, 7.390, 7.297, 7.231, 7.316, 6.883, 6.771, 6.940, 7.639, 6.871). Supplementary Table 10 compares the characteristics of the three countries of interest prior to one year before the actual event to those of the individual synthetic version. Supplementary Table 11 shows the weight of each country in the synthetic control. *Source*: the author.

**Supplementary Figure 4.** Ratio of Pre-treatment RMSPE to Post-treatment RMSPE : Chad and Control Countries

*Note*: ratio: Armenia (2.596), Bahrain (1.256), Belarus (1.324), Bhutan (1.337), Burkina Faso (2.411), Cameroon (1.190), Chad (4.420), Djibouti (4.440), Equatorial Guinea (1.681), Gabon (3.217), Gambia (4.157), Iran (3.423), Jordan (1.527), Kazakhstan (1.618), Kuwait (2.756), Laos (2.820), Mauritania (3.483), Mozambique (1.852), Singapore (2.264), Suriname (1.275), Swaziland (4.311), Tanzania (2.784), Togo (2.958), Tunisia (1.815), Turkmenistan (3.182), UAE (2.232), Uzbekistan (0.447), Vietnam (2.705). *Source*: the author.

**Supplementary Figure 5.** Health Expenditure Gap: Actual Country versus Synthetic Country

*Note*: health expenditure gap from 1996 to 2010: Chad (0.195, -0.127, -0.209, -0.211, -0.419, -0.067, 1.257, -0.870, -0.198, -2.825, -3.086, -2.371, -2.770, -2.693, -2.708), Djibouti (-0.731, -0.358, 0.332, 1.028, 0.224, 0.120, -0.088, 0.615, 1.187, 1.355, 1.370, 2.558, 2.974, 2.716, 3.356), Gambia (0.217, -0.204, 0.283, 0.326, 0.321, -0.059, -0.325, -0.279, -0.059, 0.054, -0.845, 1.061, 1.806, 1.654, 0.780), Swaziland (0.121, -0.632, -0.266, -0.241, -0.190, 0.021, -0.717, 1.199, 1.599, 2.052, 2.038, 1.794, 2.760, 2.933, 3.306). *Source*: the author.


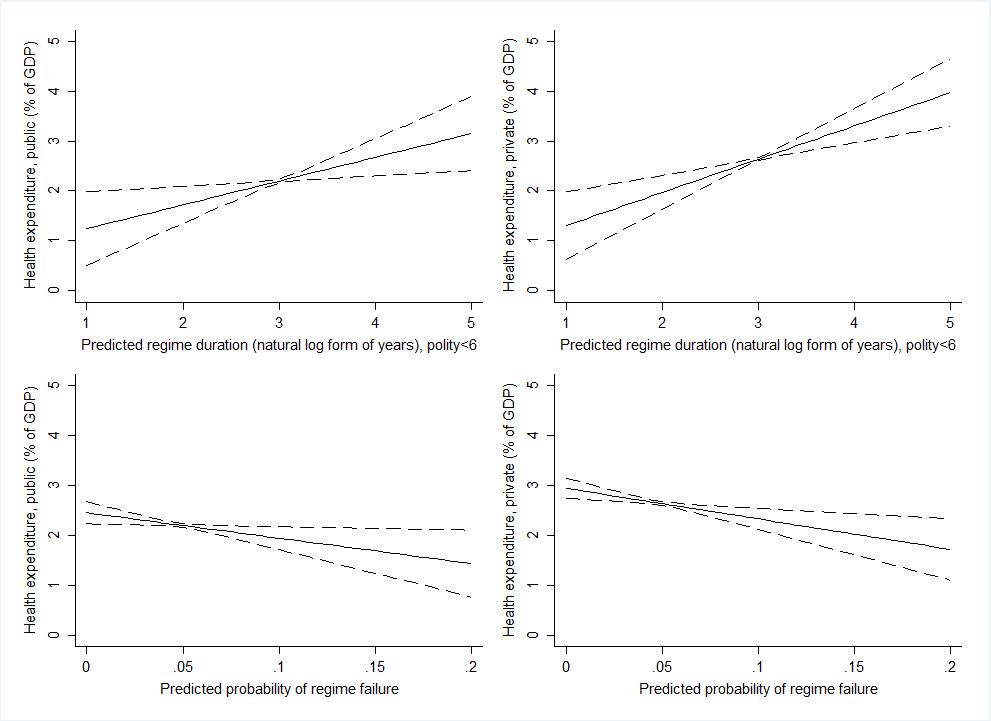


**Supplementary Figure 6.** Authoritarian Time Horizon and Health Expenditure, 1995–2010

*Note*: regression coefficient: upper-left panel: 0.476^**^, 95% CI = [0.107, 0.846]; upper-right panel: 0.667^***^, 95% CI = [0.329, 1.005]; lower-left panel: -5.077^**^, 95% CI = [-9.516, -0.637]; lower-right panel: -6.130^***^, 95% CI = [-10.198, -2.061], ^*^ *p* < 0.10, ^**^ *p* < 0.05, and ^***^ *p* < 0.01. All results were based on panel data analysis with country and time fixed effects. Public health expenditure consists of recurrent and capital spending from government (central and local) budgets, external borrowings and grants (from international donors), and social (or compulsory) health insurance funds. Private health expenditure includes direct household spending, private insurance, charitable donations, and direct service payments by private corporations. The data was from the World Bank. *Source*: the author.


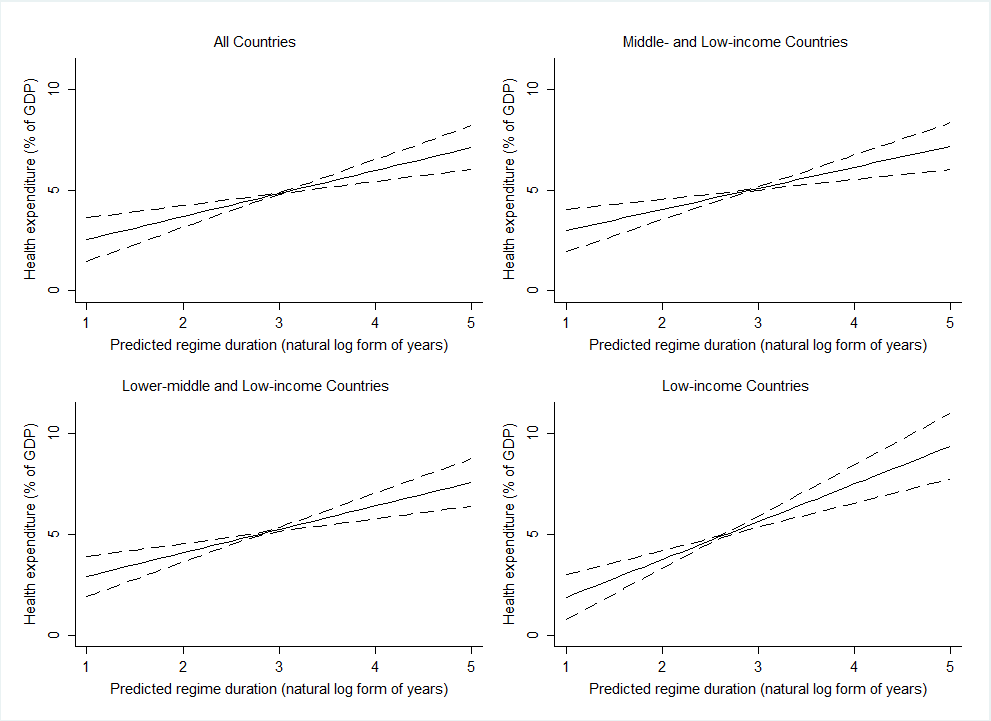


**Supplementary Figure 7.** Authoritarian Time Horizon and Health Expenditure, 1995–2010

*Note*: regression coefficient: upper-left panel: 1.143^***^, 95% CI = [0.600, 1.686]; upper-right panel: 1.050^***^, 95% CI = [0.494, 1.606]; lower-left panel: 1.167^***^, 95% CI = [0.623, 1.712]; lower-right panel: 1.880^***^, 95% CI = [1.190, 2.569], ^*^ *p* < 0.10, ^**^ *p* < 0.05, and ^***^ *p* < 0.01. All results were based on panel data analysis with country and time fixed effects. Upper-left panel: all countries; upper-right panel: middle- and low-income countries; lower-left panel: lower-middle and low-income countries; lower-right panel: low-income countries. The income classification is based on a measure of national income per person, or GNI per capita, calculated using the Atlas method. The thresholds to distinguish between the income groups have evolved over time. Please see https://datatopics.worldbank.org/world-development-indicators/stories/the-classification-of-countries-by-income.html. The data was from the World Bank. *Source*: the author.


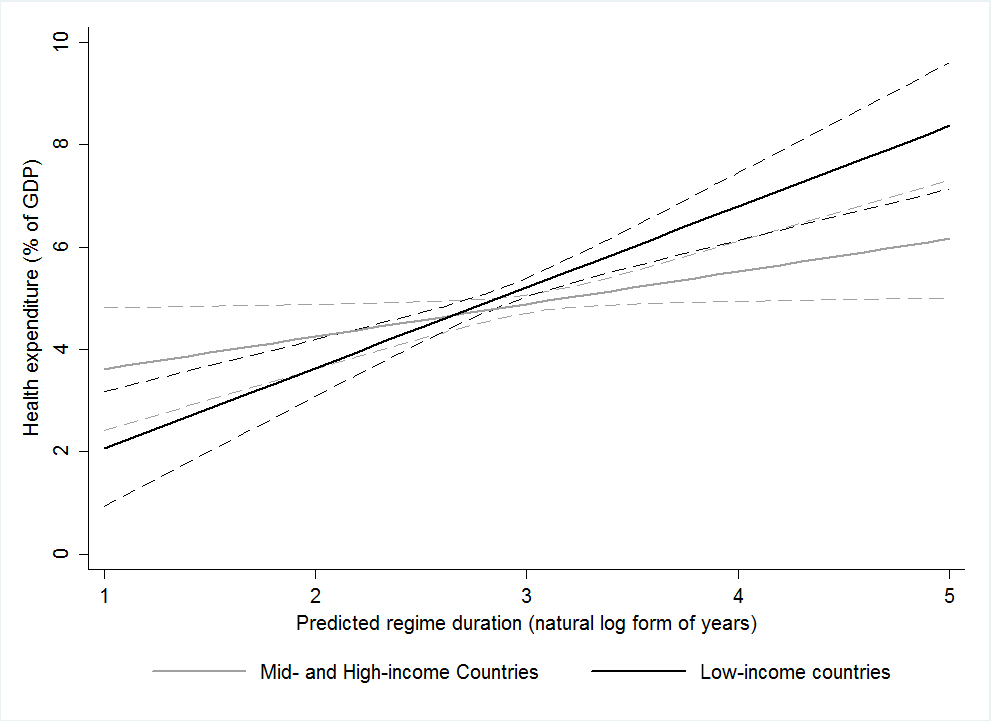


**Supplementary Figure 8.** Authoritarian Time Horizon and Health Expenditure, 1995–2010

*Note*: coefficient of the interaction term: 0.942^***^, 95% CI = [0.483, 1.401]; slope of mid- and high-income countries: 0.637^**^, 95% CI = [0.054, 1.219]; slope of low-income countries: 1.579^***^, 95% CI = [0.996, 2.161],^*^ *p* < 0.10, ^**^ *p* < 0.05, and ^***^ *p* < 0.01. The interaction term was computed: “predicted regime duration × income groups” and for income groups, where 1=low-income countries and 0=mid- and high-income countries. *Source*: the author.


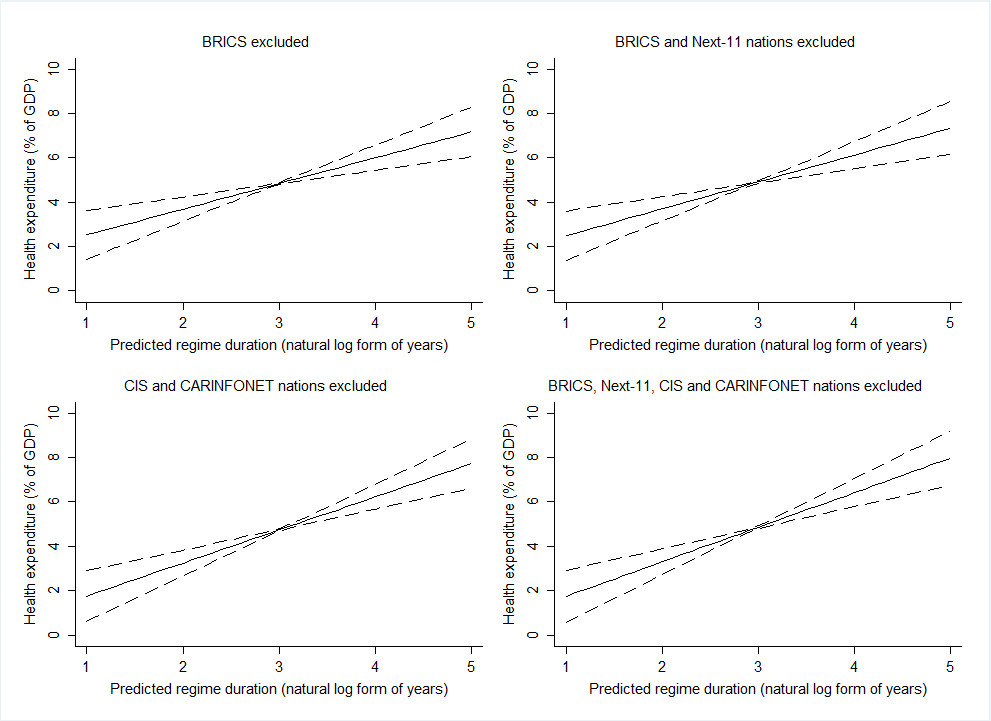


**Supplementary Figure 9.** Authoritarian Time Horizon and Health Expenditure, 1995–2010

*Note*: regression coefficient: upper-left panel: 1.161^***^, 95% CI = [0.605, 1.716]; upper-right panel: 1.215^***^, 95% CI = [0.637, 1.793]; lower-left panel: 1.494^***^, 95% CI = [0.929, 2.059]; lower-right panel: 1.560^***^, 95% CI = [0.957, 2.163], ^*^ *p* < 0.10, ^**^ *p* < 0.05, and ^***^ *p* < 0.01. All results were based on panel data analysis with country and time fixed effects. Upper-left panel: BRICS excluded; upper-right panel: BRICS and Next-11 nations excluded; lower-left panel: CIS and CARINFONET nations excluded; lower-right panel: BRICS, Next-11, CIS and CARINFONET nations excluded. BRICS: Brazil, Russia, India, China and South Africa; Next-11: Bangladesh, Egypt, Indonesia, Iran, Mexico, Nigeria, Pakistan, the Philippines, Turkey, South Korea and Vietnam; CIS: Armenia, Azerbaijan, Belarus, Kazakhstan, Kyrgyzstan, Moldova, Russia, Tajikistan, Turkmenistan, Ukraine and Uzbekistan; CARINFONET (Central Asian Republics Information Network): Kazakhstan, Kyrgyzstan, Tajikistan, Turkmenistan and Uzbekistan. *Source*: the author.

**
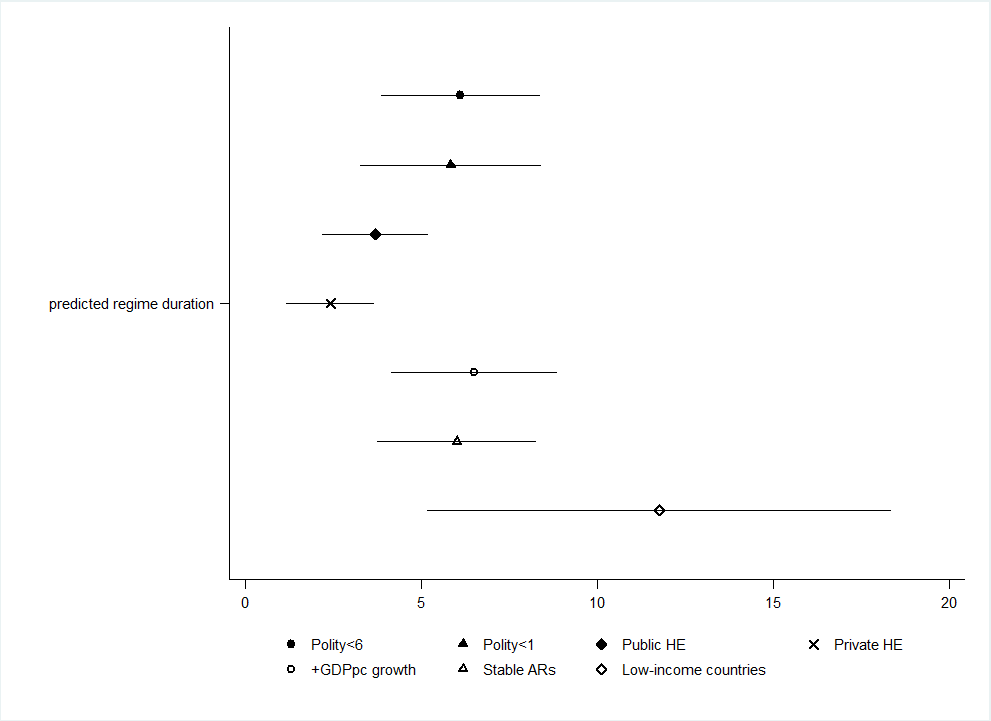
**

**Supplementary Figure 10.** IV Analysis: The Coefficient Plot of Predicted Regime Duration, 1995–2010

*Note*: HE: health expenditure; GDPpc growth: GDP per capita growth; ARs: stable authoritarian regimes. Coefficient (dictatorships as those with Polity IV scores lower than 6): 6.123^***^, 95% CI = [3.871, 8.376]; Coefficient (dictatorships as those with Polity IV scores lower than 1): 5.846^***^, 95% CI = [3.278, 8.413]; Coefficient (public health expenditure as the dependent variable): 3.700^***^, 95% CI = [2.191, 5.210]; Coefficient (private health expenditure as the dependent variable): 2.423^***^, 95% CI = [1.161, 3.686]; Coefficient (GDP per capita growth as an additional control): 6.516^***^, 95% CI = [4.167, 8.865]; Coefficient (stable authoritarian regimes): 6.022^***^, 95% CI = [3.758, 8.286]; Coefficient (low-income autocratic countries): 11.769^***^, 95% CI = [5.184, 18.353]; Weak identification test (Cragg-Donald Wald F statistic): 64.190, 49.821, 64.190, 64.190, 61.109, 61.592, 12.852. The 15% relative bias of the 2SLS estimator we are willing to tolerate is 8.96. If the Cragg-Donald F statistic exceeds the critical value, we can conclude that our instruments are not weak. The IV was the percentage of other democracies in the world. The Polity IV data series scales regimes from -10 to +10, and the study identified democracies as regimes with scores greater or equal to 6 (>=6), screening all countries between 1995 and 2010. ^*^ *p* < 0.10, ^**^ *p* < 0.05, and ^***^ *p* < 0.01. *Source*: the author.
